# Supplementary material for: Systematic analysis of functional genetic and epigenetic variants in colorectal cancer
Source: Sci Adv. 2026 Feb 20;12(8):eaeb2473. doi: 10.1126/sciadv.aeb2473 (PMC12922747; doi:10.1126/sciadv.aeb2473)
Supplement: 20260220-1 [file sciadv.aeb2473.v1.pdf]

CANCER

Copyright © 2025 The Authors, some rights reserved; exclusive licensee American Association for the Advancement of Science. No claim to original U.S. Government Works. Distributed under a Creative Commons Attribution License 4.0 (CC BY).

Systematic analysis of functional genetic and epigenetic variants in colorectal cancer

Erfei Chen[1], Qiqi Yang[1]†, Haoyang Dai[1]†, Yixin Chen[1]†, Yihui Zhang[1]†, Qianglong Wang[1]†, Rongrong Hou[1], Ming Chen[1], Jie Wang[1], Qianwen Xie[1,2,3], Wenju Sun[1], Yong-Qiang Ning[1], Ligang Fan[1], Jian Yan[1,2,3,4]*

Colorectal cancer (CRC) is a leading cause of cancer-related mortality worldwide, yet the functional impact of noncoding variants on enhancer activity remains largely unexplored. In this study, we adapted and applied two high-throughput techniques, SNP-STARR-seq and Methyl-STARR-seq, to systematically evaluate the influence of 30,790 noncoding SNPs and more than 134,000 CpG sites on enhancer activity in primary and metastatic CRC cells. We identified 922 SNPs and 487 CpG-containing elements modulating enhancer activity in primary cells and found 3136 SNPs and 3008 methylation-sensitive elements with metastasis-specific regulatory effects. Multi-omics integration linked these variants to target genes, and CRISPR editing validated their roles in driving tumorigenic and metastatic phenotypes. Furthermore, we identified two CRC-specific hypermethylated loci, cg08640619 and cg25982657, as exceptional tissue-based early detection biomarkers (AUC > 0.96). Mechanistically, hypermethylation at cg08640619 disrupts RUNX2 binding, leading to inhibition of *KIRREL1* and *ETV3*. Our study provides a comprehensive platform for understanding how genetic and epigenetic variants disrupt transcriptional programs in CRC, offering insights into disease susceptibility and identifying potential diagnostic and therapeutic targets.

INTRODUCTION

Colorectal cancer (CRC) is a genetically complex malignancy driven by cumulative genomic and epigenomic alterations. While coding mutations (e.g., *APC* and *KRAS*) are well-characterized drivers (*1*), more than 90% of disease-associated variants from genome-wide association studies (GWASs) reside in nonprotein-coding regions (*2*), suggesting critical roles for regulatory element dysregulation. Enhancers, in particular, coordinate spatiotemporal gene expression through chromatin looping (*3*), and their perturbation by single-nucleotide polymorphisms (SNPs) or DNA methylation contributes to oncogenesis (*4*).

Enhancers exhibit profound tissue and cell-type specificity in their activity patterns, a property fundamental to precise spatiotemporal gene regulation during development and disease (*3*). In cancer, this specificity drives tumor-context–dependent oncogenic programs, where identical genetic variants may exert divergent effects across malignancies (*5*). More than 70% of cancer risk variants show tissue-specific enhancer effects (*6*). For instance, one of the best-studied CRC risk SNP rs6983267 was mapped to a super-enhancer at 8q24, a "gene desert" locus located ~335 kb upstream from the promoter of the proto-oncogene *MYC* (*7*). Its "G" allele recruited binding of transcription factor (TF) TCF7L2 to amplify Wnt-driven oncogenesis. This landmark study established the paradigm of noncoding SNP-regulating proto-oncogenes through chromatin long-range interactions. Notably, in pancreatic cancer and AML cells, functional enhancers reside to the downstream of *MYC* gene instead. This finding underscores the tissue-specific nature of enhancer function and the regulatory impact of variants within these elements (*4*). Recent studies reveal that CRC progression also involves dynamic epigenetic reprogramming of enhancers. Metastatic transition is marked by widespread enhancer hijacking (*8*), while aberrant methylation at enhancer-associated CpG islands silences tumor suppressors (*9*). Functional genomics approaches, such as massively parallel reporter assay (MPRA) in primary cells (*10*) and silencer-activity–related variants screening (*11*), have provided invaluable insights into molecular function of the noncoding genetic variants. However, systematic annotation of regulatory function of noncoding variants and differentially methylated regions (DMRs) in CRC enhancers, especially in metastatic contexts, remains limited, in that traditional methods like reporter assays generally lack scalability.

In this study, we first performed pan-cancer analysis of active enhancer regions across 20 malignancies using assay for transposase-accessible chromatin using sequencing (ATAC-seq) and H3K27ac chromatin immunoprecipitation sequencing (ChIP-seq) data. This comparative framework enabled systematic discrimination of common regulatory variants with pan-cancer impact from tumor-specific alterations unique to CRC pathogenesis, thereby prioritizing context-dependent drivers for functional screening. For CRC enhancers, we captured corresponding regulatory SNPs and CpG methylation sites. Subsequently, we designed and synthesized high-complexity oligo libraries that enclosed 120–base pair (bp) genomic fragments surrounding the SNPs or CpG sites of interest. Leveraging two high-throughput strategies, SNP self-transcribing active regulatory region sequencing (SNP-STARR-seq) (*12*, *13*) and methylation self-transcribing active regulatory region sequencing (Methyl-STARR-seq) (*14*), in CRC cells including two primary CRC-derived epithelial cell lines HCT116 and SW480, as well as a metastatic CRC-derived SW620 cell line of the same origin of patient as SW480, we systematically quantified the impact of genetic and epigenetic variants in enhancer activity and identified 922 common SNPs with significant enhancer activity alterations shared between primary cell lines (HCT116 and SW480), alongside

[1]College of Life Sciences and School of Medicine; Ministry of Education Key Laboratory of Resource Biology and Biotechnology in Western China, Northwest University, Xi'an 710069, China. [2]Department of Biomedical Sciences, Tung Biomedical Sciences Centre, College of Biomedicine, City University of Hong Kong, Kowloon Tong, Hong Kong. [3]Department of Precision Diagnostic and Therapeutic Technology, The City University of Hong Kong Shenzhen Research Institute, Shenzhen 518057, China. [4]Department of Urology, The First Affiliated Hospital of Xi'an Jiaotong University, Xi'an 710061, China.
*Corresponding author. Email: jian.yan@cityu.edu.hk
†These authors contributed equally to this work.

3136 metastasis-acquired regulatory SNPs unique to SW620. Similarly, Methyl-STARR-seq revealed 1763 and 3588 methylation-sensitive enhancer elements in HCT116 and SW480, respectively, and 4112 in SW620, of which 3008 were specifically dysregulated in the metastatic context. Through multi-omics integration [Hi-C, expression quantitative trait locus (eQTL), and RNA sequencing (RNA-seq)], we linked these variants to their regulatory target genes.

Unraveling the molecular function of these variants, we showed that the variant rs67941642 [a SNP in high linkage disequilibrium (LD) with the GWAS lead SNP rs6061231 on chromosome 20q] could dominantly enhance TF growth factor independent 1 transcriptional repressor (GFI1) binding and promote expression of the *TAF4* tumor suppressor gene, whose down-regulation drove CRC cell proliferation and migration. For metastasis-associated variants, we also revealed that "A" allele of SNP rs1962004, which was acquired somatically in the metastatic SW620 line, created a binding site for the activator protein 1 (AP-1) component TFs Jun proto-oncogene, AP-1 transcription factor subunit (JUN) and JunD proto-oncogene, AP-1 transcription factor subunit (JUND), activating the *LRRC61* oncogene to promote metastatic phenotypes and correlating with poor patient prognosis. In terms of CRC-risk related methylation sites, we found that hypermethylation at cg08640619 disrupted the binding of the TF RUNX2, leading to down-regulation of the nearby genes *KIRREL1* and *ETV3*. Crucially, both cg08640619 demonstrated exceptional performance as early-detection biomarkers [area under the receiver operating characteristic curve (AUROC) > 0.96]. Our study introduces a powerful experimental pipeline to functionally screen for regulatory genetic and epigenetic variants.

RESULTS

Project design to identify functional variants in primary and metastatic CRC

How context-dependent regulatory variants drive CRC pathogenesis, particularly during metastasis remained poorly captured in prior studies. Here, we developed a strategy that was able to systematically evaluate both genetic and epigenetic variants for their impact in the transcriptional regulatory activity of the CRC enhancer elements (Fig. 1).

First, we defined a set of putative CRC enhancer regions by identifying 18,880 genomic intervals that overlapped between H3K27ac ChIP-seq and ATAC-seq peaks in the CRC cell line HCT116, indicating both active regulatory activity and chromatin accessibility. To distinguish the CRC regulatory landscape from common pan-cancer elements, we performed a comparative epigenomic analysis across 20 malignancies (tables S1 and S2), which allowed us to classify these enhancers as either common across many cancers or more restricted in their activity profile.

Subsequently, we used two distinct systems, SNP-STARR-seq and Methyl-STARR-seq, to assess the enhancer activity that contained the genetic and epigenetic variations, respectively. For the SNP-STARR-seq library, we synthesized a 120-nucleotide (nt) genomic library encompassing all SNP sites within 18,880 putative enhancer regions and GWAS-hit and GWAS-linked SNP loci. For each SNP, a 120-bp genomic sequence centered on the variant was included. All possible allelic variants (A, T, C, and G) at the polymorphic site were synthesized as individual oligonucleotides, enabling a comprehensive assessment of allele-specific effects for known human genetic variants. For the methylation library, we focused on two categories of genomic sequences: (i) 120-nt windows centered at H3K27ac peak summits, as these active enhancer and promoter regions are established hotspots for epigenetic reprogramming in cancer, where DNA methylation changes exert their most profound functional impact. (ii) genomic regions corresponding to CRC DMRs. Following plasmid library construction, we performed in vitro enzymatic treatment using methyltransferase M.SssI to generate two distinct libraries: fully methylated plasmid library (M.SssI treated) and unmethylated control plasmid library (mock treated) (see Materials and Methods). The enhancer activity scores (EAS) were derived from DESeq2-normalized RNA/DNA read counts as EAS = (RNA + 1)/DNA. Allelic or methylation

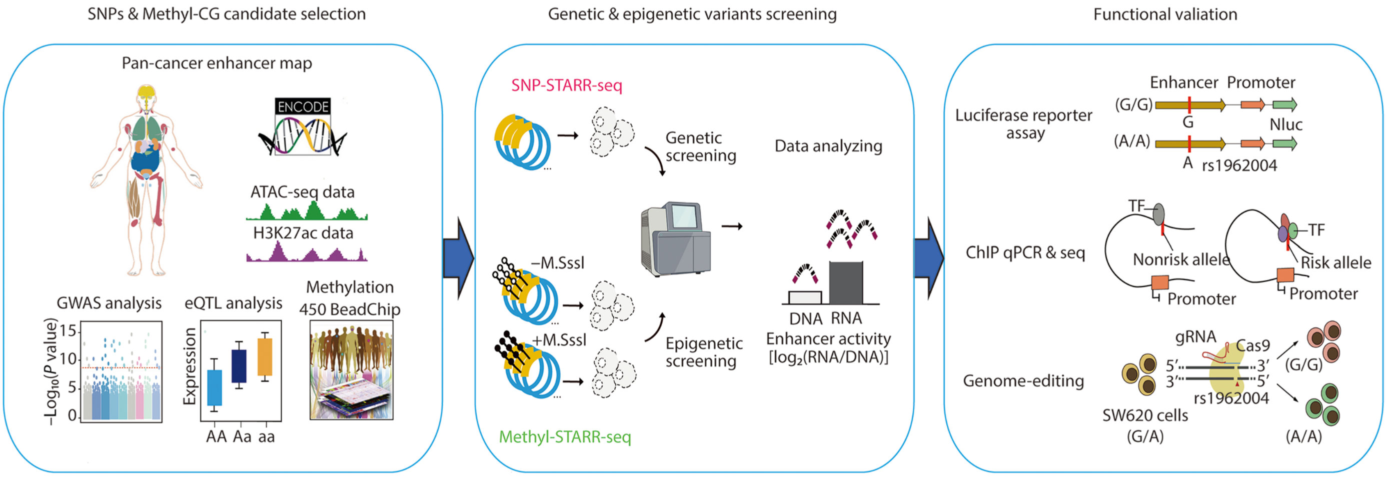

Fig. 1. Integrated identification and validation of functional genetic and epigenetic variants in enhancer elements. This schematic outlines a comprehensive approach for identifying and validating functional variants in enhancer regions across cancer types. (i) Candidate selection: SNPs and methylated CpG (Methyl-CG) sites are prioritized using a pan-cancer enhancer map derived from ATAC-seq (chromatin accessibility) and H3K27ac (active enhancer) data. Using GWAS, methylation profiling, and eQTL data, we identified trait-associated variants and epigenetic variants and linked them to target gene expression. (ii) Functional screening: SNP-STARR-seq and Methyl-STARR-seq were used to assess allele-dependent and methylation-dependent enhancer activity, respectively. (iii) Validation: We validated how variants affect TF binding and downstream transcriptional regulation using luciferase reporter assays, ChIP assays, and tumor phenotype assays after CRISPR-Cas9 editing, as exemplified by the metastasis-associated variant rs1962004.

fold changes were calculated by comparing EAS values between alternative/reference or methylation/unmethylation, with statistical significance. Through systematic screening in three CRC cell lines, primary adenocarcinoma models (HCT116 and SW480) and a metastatic derivative cell line (SW620), we identified functional regulatory variants significantly modulated by both genetic mutations and epigenetic alterations. Preferentially active SNPs (paSNPs; defined as SNP conferring significantly differential enhancer score and adjusted $P < 0.05$; see Materials and Methods) and preferentially active methylation sites (paMethylsites; defined as CpG methylation conferring significantly differential enhancer score and adjusted $P < 0.05$; see Materials and Methods) identified across three cell lines are presented in tables S3 and S4.

By further integrating clinically relevant RNA-seq, eQTL, and epigenomic datasets, we assigned the regulatory variants to their target genes. Focusing on variants implicated in CRC development and metastasis, we validated the functional relevance of the identified variants obtained through this screening pipeline using techniques including reporter gene assays, chromatin immunoprecipitation (ChIP), and CRISPR-based genome editing. Collectively, this integrated functional genomics framework not only deciphers the mechanistic links between noncoding variants and CRC pathogenesis/metastasis but also pinpoints previously unidentified therapeutic targets and highly sensitive diagnostic biomarkers with significant translational potential.

SNP-STARR-seq identified SNPs conferring differential enhancer activities in CRC cells

Within 18,880 candidate enhancers, we selected SNPs with a minor allele frequency (MAF) ≥ 0.05 in both European and East Asian populations from the 1000 Genomes Project (*15*), resulting in 24,526 common SNPs. To investigate the regulatory potential of SNPs within enhancer regions, we incorporated data from the GWAS Catalog and retrieved 238 genome-wide significant CRC-associated loci, along with 6125 additional SNPs in LD [coefficient of determination (R^2) ≥ 0.5] with these loci. When combining the SNPs identified from CRC enhancer regions with the GWAS-related SNPs, we obtained 30,790 unique SNPs as candidate regulatory elements for functional screening analysis. For each SNP, a 120-bp genomic sequence centered on the variant (±60 bp) was extracted. All allelic variants were synthesized as individual oligonucleotides. In total, we designed and synthesized 91,566 unique oligonucleotides, forming a comprehensive CRC SNP-STARR-seq library for massively parallel reporter assay (Fig. 2A). Hierarchical clustering demonstrated high reproducibility across both biological and technical [polymerase chain reaction (PCR)] replicates of the same cell type (Fig. 2B). For each SNP, the allele-specific effect on enhancer activity was quantified by calculating the logarithm-transformed change of EAS between the alternative allele and the reference allele. All qualified SNPs were then ranked on the basis of this value, and SNPs with statistically significant differential enhancer activities were selected for subsequent functional characterization (HCT116, $n = 1969$ SNPs; and SW480, $n = 1846$ SNPs; Fig. 2C and table S3). Validation of SNP-STARR-seq hits using single-locus luciferase reporter assay demonstrated strong concordance with high-throughput sequencing results across both cell lines (Fig. 2D and fig. S1A).

Both HCT116 and SW480 cell lines represent early-stage colorectal adenocarcinoma, which exhibited significant overlapping of functional variants ($P = 0.048$, Fisher's exact test; Fig. 2E). Then, we focused on 922 common SNPs exhibiting significant effects in both cell lines (table S5). Function of these noncoding variants is also supported by eQTL data (82.65%), demonstrating their regulatory impact on proximal or distal gene expression (Fig. 2F). Additionally, more than 75% of these variants were computationally predicted to harbor TF-binding sites (Fig. 2G). Similar results were obtained from analyses of each cell line individually (fig. S1, B and C). Hypergeometric Optimization of Motif EnRichment (HOMER) motif analysis further revealed significant enrichment of CRC-associated TFs within the genomic segments showing significant allelic enhancer activity differences, including basic leucine zipper ATF-like transcription factor (BATF), FOS like 2, AP-1 transcription factor subunit (FOSL2), and Fos-related antigen-2 (FRA2), which have been directly implicated in CRC progression and metastasis (*16*, *17*), along with JUNB, a component of the AP-1 complex known to be dysregulated in CRC (Fig. 2H and fig. S1D) (*18*). Based on our aforementioned pan-cancer enhancer annotation, 81.6% of the 922 common SNPs resided within CRC putative enhancer regions, although some GWAS-linked variants fall outside these regions. This enrichment is significantly higher than that observed in 19 other cancer types, confirming that our high-throughput system effectively identified regulatory variants relevant to the CRC context (Fig. 2I). Collectively, these findings demonstrated that the SNP-STARR-seq could successfully capture the vast majority of functional variants.

The epistatic role of SNP rs67941624 in driving enhancer activity in CRC cells

Among the 922 putatively functional SNPs, rs6061231 is located in a CRC risk locus and captured as a lead SNP by GWAS (*19*), while SNP rs67941642 is in complete LD ($R^2 = 1$, https://ldlink.nih.gov/) with rs6061231 (Fig. 3A). Both SNPs resided within the genomic regions bearing strong enhancer-associated epigenetic marks (H3K4me1 and H3K27ac) in human CRC cells (Fig. 3B). Given the close locations and genetic linkage, we used a dual-luciferase reporter assay to investigate the synergistic role of the two variants. First, we confirmed the enhancer activity of genomic fragments containing either the major or minor alleles of SNPs rs6061231 and rs67941642. Notably, the rs67941642-T allele exhibited significantly stronger enhancer activity than the C allele, and SNP rs6061231-C allele showed significantly stronger activity than the A allele (Fig. 3B), consistent with SNP-STARR-seq result (table S5). In the combined East Asian and European (EAS + EUR) cohort, haplotype analysis of the rs6061231/rs67941642 locus revealed frequencies of 77.0% for the C-C haplotype (rs6061231-C/rs67941642-C) and 23.0% for the A-T haplotype (rs6061231-A/rs67941642-T), whereas the other haplotypes (i.e., C-T and A-C) virtually never occur in population. Because the two LD SNPs exhibited opposite enhancer activity, we asked which SNP was dominant in determining the allelic enhancer activity. Therefore, we constructed the reporter plasmids containing the C-C and A-T haplotypes, respectively, and transfected them to CRC cells for quantitative comparison. The assay result showed that the A-T haplotype exhibited significantly higher enhancer activity compared to the C-C haplotype ($P < 0.001$, Fig. 3C), which indicated that the SNP rs67941642 conferred a predominant role in modulating the enhancer activity within its genomic context. SNP rs6061231 was a lead SNP in GWAS instead, suggesting that the lead SNP in fact turned out a proxy to the functional variant of SNP rs67941642. While both SNPs exhibited allele-specific enhancer activity, the A-T haplotype (rs6061231-A/rs67941642-T) conferred significantly stronger activity

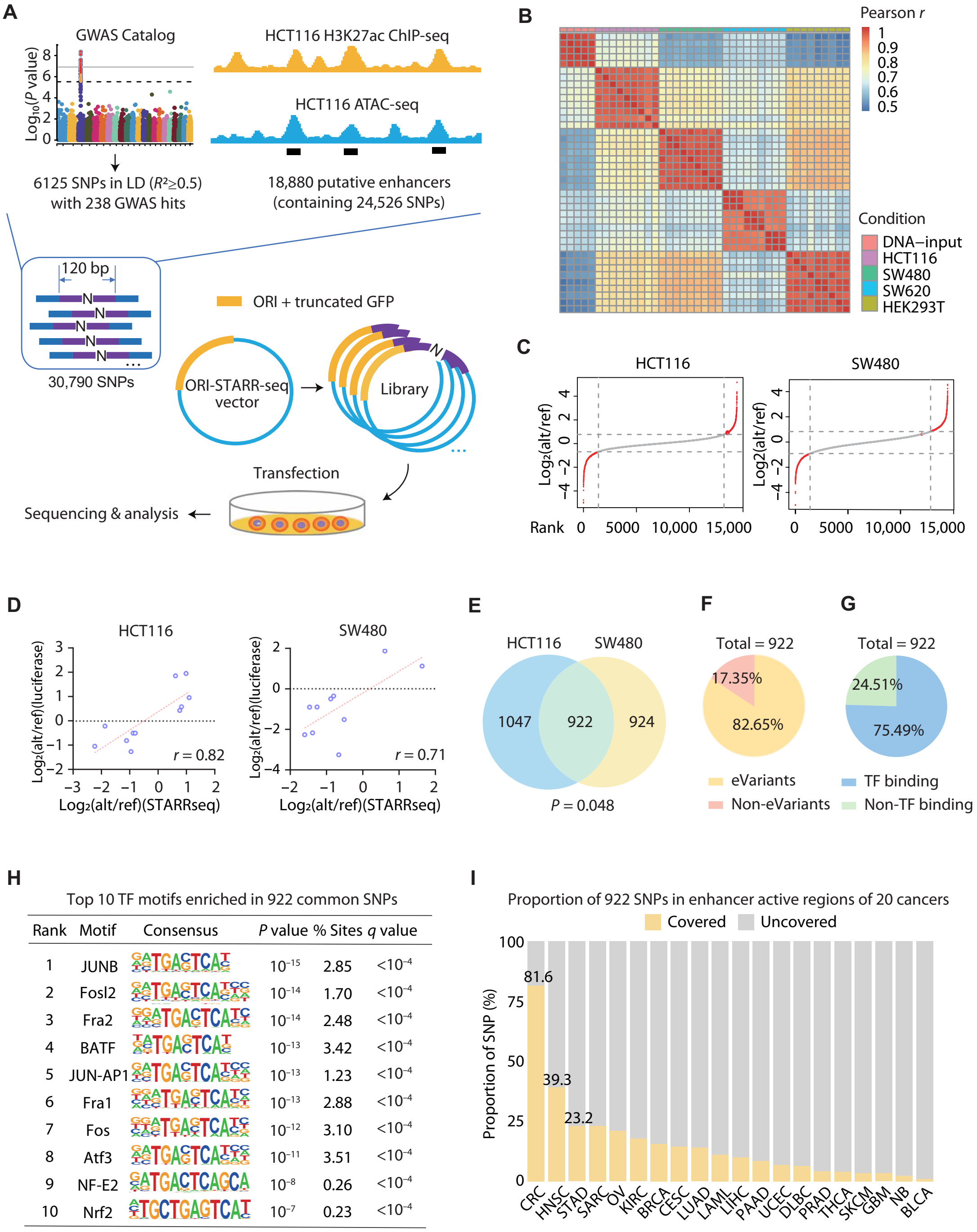

Fig. 2. SNP-STARR-seq identifies SNPs with allele-specific enhancer activities in different types of CRC cells. (**A**) Designing a STARR-seq library to screen CRC variants and overview of SNP-STARR-seq workflow. (**B**) Heatmap displaying Pearson correlation coefficients (r) of normalized read counts between all biological and technical replicates for both DNA input and RNA output libraries. High correlation among DNA input replicates reflects technical reproducibility. Clustering of RNA replicates by cell type indicates robust, cell-type–specific capture of enhancer activity. (**C**) Ranking of the $\log_2$ fold change ($\log_2$FC) of alt/ref EAS. The dashed lines show the cut off of significant paSNP (HCT116: left, −0.63; and right, 0.71; SW480: left, −0.89; and right, 0.79). The representative results of three biological repeats in HCT116 and SW480 cells were shown. (**D**) Luciferase assay validation of paSNP tested by STARR-seq. Each point represents the average of three STARR-seq and four Luciferase replicates. (**E**) The Venn diagram shows the intersection of HCT116 and SW480 significant paSNPs. The P value for the overlap of significant SNPs was calculated using Fisher's exact test. (**F**) The pie charts display the percentage of common SNPs with eQTL data. (**G**) The pie charts display the percentage of common SNPs with TF binding. (**H**) The top 10 TF motif enriched in 922 common SNPs. (**I**) The proportion of 922 CRC-associated SNPs located within enhancer active regions across 20 different cancer types. Enhancer active regions were defined as the intersection of H3K27ac ChIP-seq and ATAC-seq or DNase-seq enrichment peaks. Yellow bars represent SNPs covered by enhancer regions, while gray bars indicate uncovered SNPs. The results showed that, while 81.6% (752 of 922) of these SNPs resided within CRC enhancers, substantially lower proportions were found in enhancer regions of other cancer types [e.g., 39.3% in head and neck squamous cell carcinoma (HNSC) and 23.2% in stomach adenocarcinoma (STAD)], suggesting a tissue-specific enrichment of these SNPs in CRC enhancer elements.

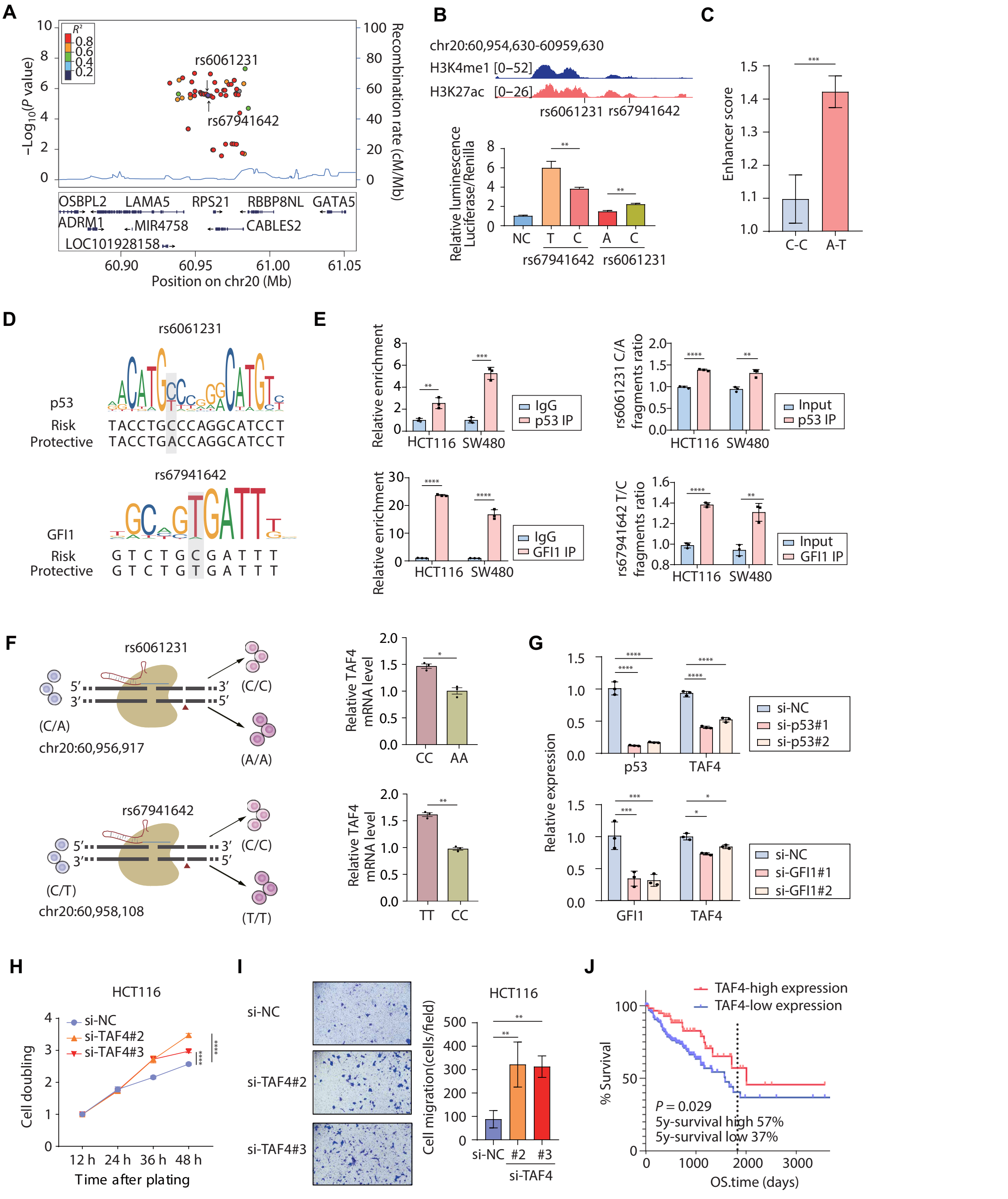

Fig. 3. Functional characterization of GWAS-risk SNP rs6061231 and rs67941642. (**A**) Regional association plot for the 21q13.3 locus identified in GWAS meta-analysis. (**B**) The two SNP loci locate in the enhancer region marked by H3K4me1 and H3K27ac signals. The luciferase assays show an allele-specific enhancer activity at both rs6061231 and rs67941642. (**C**) EAS analysis of common human haplotypes (rs6061231-C/rs67941642-C versus rs6061231-A/rs67941642-T). (**D**) Motif analysis of p53 at rs6061231 and GFI1 at rs67941642. (**E**) ChIP–quantitative polymerase chain reaction (qPCR) for p53 binding at the rs6061231 and GFI1 binding at the rs67941642 in both HCT116 and SW480 cells (left). Allele-specific binding of p53 to rs6061231-C and GFI1 to rs67941642-T was assessed by ChIP-amplicon sequencing analysis (right). IP, immunoprecipitation. (**F**) CRISPR-Cas9–mediated single variant mutation of rs6061231 and rs67941642 in HCT-116 cells, generating homozygous cell lines, respectively. qPCR analysis of *TAF4* expression in each mutated cell line (paired t test). (**G**) Effect of p53 or GFI1 knockdown on *TAF4* expression. (**H**) Effect of *TAF4* knockdown on HCT116 cell proliferative capacity (assessed by CCK-8 assay). h, hours. (**I**) Effect of *TAF4* knockdown on HCT116 cell migration (Transwell assay). (**J**) Kaplan-Meier analysis of TAF4 expression in TCGA-COAD cohort. 5y, 5 years; *$P < 0.05$, **$P < 0.01$, ***$P < 0.001$, and ****$P < 0.0001$.

than the C-C haplotype (Fig. 3C). This indicates that the net regulatory output of this locus is dominated by the combined effect of both SNPs, with rs67941642-T playing a predominant role in determining the overall enhancer strength of the common human haplotypes. It has been established that some genes exhibited an epistatic role over other genes in expressing the phenotype. Here, we revealed epistatic regulatory elements that displayed a dominant role over other enhancers in regulating the transcription of the target gene. The result further underscored the importance of comprehensively analyzing the molecular function of variants in LD and in adjacent to GWAS hits.

Previous studies have demonstrated that numerous functional noncoding genetic variants can alter TF binding. The motif analysis suggested specific binding of TFs GFI1 to rs67941642-T, p53 to rs6061231-C, and SIX homeobox 2 (SIX2) to rs6061231-A (Fig. 3D and fig. S2A). Genotyping confirmed heterozygosity of both SNPs rs67941642 and rs6061231 in HCT116 and SW480 cells (fig. S2B). Subsequent ChIP–quantitative polymerase chain reaction (qPCR) and ChIP-amplicon sequencing revealed preferential recruitment of GFI1 to rs67941642-T ($P < 0.001$) and p53 to rs6061231-C ($P < 0.001$), while SIX2 binding showed no significant allelic bias at rs6061231 (Fig. 3E and fig. S2C). Given the contrasting effects of both SNPs on enhancer activity, we investigated whether the two TFs might have an interfering role at this locus. Thus, we individually knocked down p53 and GFI1 in HCT116 cells and found that the binding of GFI1 at rs67941642 or p53 at rs6061231 was not significantly affected, respectively, suggesting that no mutual influence between these two TFs existed (fig. S2D).

The rs6061231/rs67941642 locus cis-regulated *TAF4* expression

A previous study had shown that the copy number amplification of 20q served as a prognostic biomarker in the microsatellite stable subtype of CRC (*20*). Because the SNPs rs6061231 and rs67941642 are located within the Chr20q locus, we envisioned to understand the relevance to the clinical significance. First, we intended to unravel the targeted genes of the SNP-containing elements. By analyzing the Hi-C data of HCT116 cells and focusing on tumor-associated differentially expressed genes (DEGs) within the same topologically associating domain (TAD), a few candidate genes were consequently identified, including *TAF4*, *CABLES2*, *MTG2*, *RBBP8NL*, *ADRM1*, *DIDO1*, *LINC00659*, *RPS21*, *YTHDF1*, and *HAR1A*. To fine-map their regulatory relationship, we generated two independent knockout (KO) HCT116 cell lines using CRISPR-Cas9, each with a short fragment (89 bp and 136 bp, respectively) spanning the target SNP rs6061231 removed from the genome. When assessing the impact of the above candidate genes, only *TAF4* and *LINC00659* showed significant changes of expression in both KO clones (fig. S2E). Our data had demonstrated that the region around rs6061231 conferred enhancer activity to activate the target gene expression (Fig. 3B), whereas deletion of the element led to up-regulation of *LINC00659*, excluding the direct regulatory relationship between them. In contrast, *TAF4* expression was markedly reduced ($P < 0.001$) upon enhancer deletion (fig. S2F), backing the possibility that *TAF4* was the actual target of SNP rs6061231.

To further consolidate the allele-specific regulation of *TAF4* by SNP rs6061231, we generated homozygous C/C and A/A genotypes of HCT116 cell clones through CRISPR-Cas9 editing. The qPCR analysis showed significantly elevated *TAF4* expression ($P < 0.001$) in homozygous C/C cells compared to that in A/A cells. Similarly, homozygous rs67941642 T/T cells also exhibited significantly higher *TAF4* expression than C/C cells ($P < 0.05$) (Fig. 3F). These results were highly consistent with the enhancer activity assay (Fig. 3B), demonstrating the allele-specific regulation of *TAF4* by both rs6061231 and rs67941642. The expression of *LINC00659*, *MTG2*, *DIDO1*, *RBBP8NL*, and *OSBPL2* was not significantly altered by the single nucleotide changes (assessed by paired *t* test, fig. S2G). This result, combined with our H3K27ac ChIP-seq data showing enhanced enhancer activity at the *LINC00659* locus upon deletion (fig. S2H), indicates that the enhancer directly and specifically regulates *TAF4* and that the up-regulation of *LINC00659* is an indirect consequence of structural perturbations rather than a direct regulatory relationship. To assess the role of TFs GFI1 and p53 bound to the two SNPs in regulating *TAF4* expression, we performed knockdown experiments targeting these TFs. Depletion of either GFI1 or p53 significantly down-regulated *TAF4* expression (Fig. 3G). These results established that the enhancers harboring these SNPs activated *TAF4* transcription through recruitment of GFI1 and p53.

Previous studies have found that murine Taf4 antagonizes PRC2-mediated inhibition of stem cell gene expression to maintain intestinal homeostasis (*21*). To probe the tumor suppressor function of *TAF4*, we performed *TAF4* knockdown in HCT116 cells using small interfering RNA (siRNA; fig. S2I). Lack of *TAF4* significantly promoted both cell proliferation (Fig. 3H) and migration (Fig. 3I), reflecting the malignant phenotypes. To mechanistically link this growth-suppressive function directly to the enhancer activity, we analyzed the proliferation of the enhancer-KO cells. Consistent with the loss of a growth-inhibitory regulator, these enhancer-KO cells exhibited enhanced cell proliferation (fig. S2J). We further assessed the phenotypic consequence of the allele-specific regulatory effects by performing proliferation assays on the homozygous point-mutated cell lines. For rs67941642, cells with the T/T genotype (associated with higher *TAF4* expression) showed a trend toward reduced proliferation compared to C/C cells, aligning with the tumor-suppressive role of *TAF4*. In contrast, for rs6061231, switching from A/A to C/C genotype did not result in a statistically significant difference in cell growth.

The survival analysis using The Cancer Genome Atlas Program (TCGA) data revealed that patients with low expression (37%) of *TAF4* suffered a significantly poorer 5-year overall survival rate (57%) compared to those with high *TAF4* expression ($P < 0.05$, Fig. 3J). These findings established that *TAF4* was a tumor suppressor under the regulation of two highly connected CRC risk SNPs rs6061231/rs67941642, and the allele with overall synergistically lower enhancer activity was associated with poor clinical outcome. As we discussed above, the regulatory activity of lead SNP rs6061231 was dominated by rs67941642, and the risk C-allele of rs6061231 associated with the lower expression of *TAF4* actually conferred stronger enhancer activity.

SNP-STARR-seq discovered SNPs potentially contributing to CRC metastasis

Metastasis is a leading cause of death in patients with CRC, driven by complex molecular alterations acquired during tumor progression. To systematically identify functional genetic variants that contributed to the metastatic transition, we leveraged unique isogenic cell model, comparing regulatory activity of SNPs in SW480 (a primary CRC-derived epithelial cell line) and SW620 (derived from a lymph node metastatic tumor of the same patient with CRC as

SW480). This paired cell model provided an exceptionally powerful system to pinpoint metastasis-acquired genetic and epigenetic changes, minimizing confounding interindividual genetic background differences. We hypothesized that loci exhibiting function in driving enhancer activity specifically in the metastatic SW620 cells, but not in SW480 cells, represented potential genetic or epigenetic factors associated with metastatic traits.

Using the SNP-STARR-seq system, we screened for SNPs exhibiting allele-specific differences in enhancer activity between SW480 and SW620 cell lines (table S3). The paSNPs identified in SW620 cells were predominantly functional variants, with 88.9% supported by eQTL evidence and 72.3% predicted to reside within TF-binding sites (fig. S3A). Cross-reference of the results from the two cell lines identified 3136 SNPs that exhibited allele-specific enhancer activity specifically in SW620 cells (Fig. 4A and table S6). HOMER analysis predicted significant enrichment of binding motifs for bZIP TFs around these SNPs (Fig. 4, B and C), a TF structural family predominantly associated with CRC metastasis (*16*, *22*–*25*).

We then sought to further refine the mapping of functional genetic variants and loci contributing to CRC metastasis. RNA-seq analysis comparing SW480 and SW620 cells identified 2560 genes that were significantly and specifically up-regulated in SW620, suggesting their strong association with CRC metastasis [adjusted $P < 0.05$, $\log_2$ fold change ($\log_2$FC) > 0.75; fig. S3B and table S7]. Functional enrichment analyses [Gene Ontology (GO) and Kyoto Encyclopedia of Genes and Genomes (KEGG)] confirmed that these up-regulated genes were closely linked to metastasis-related processes, such as cell migration and Wnt signaling pathways (fig. S3C). Among the 3136 SW620-specific regulatory SNPs, 2517 (80.3%) were located within ±1 Mbp of the transcription start sites of these metastasis–up-regulated genes, suggesting a regulatory relationship between the genetic variants and these genes. By integrating these findings with SW620-specific

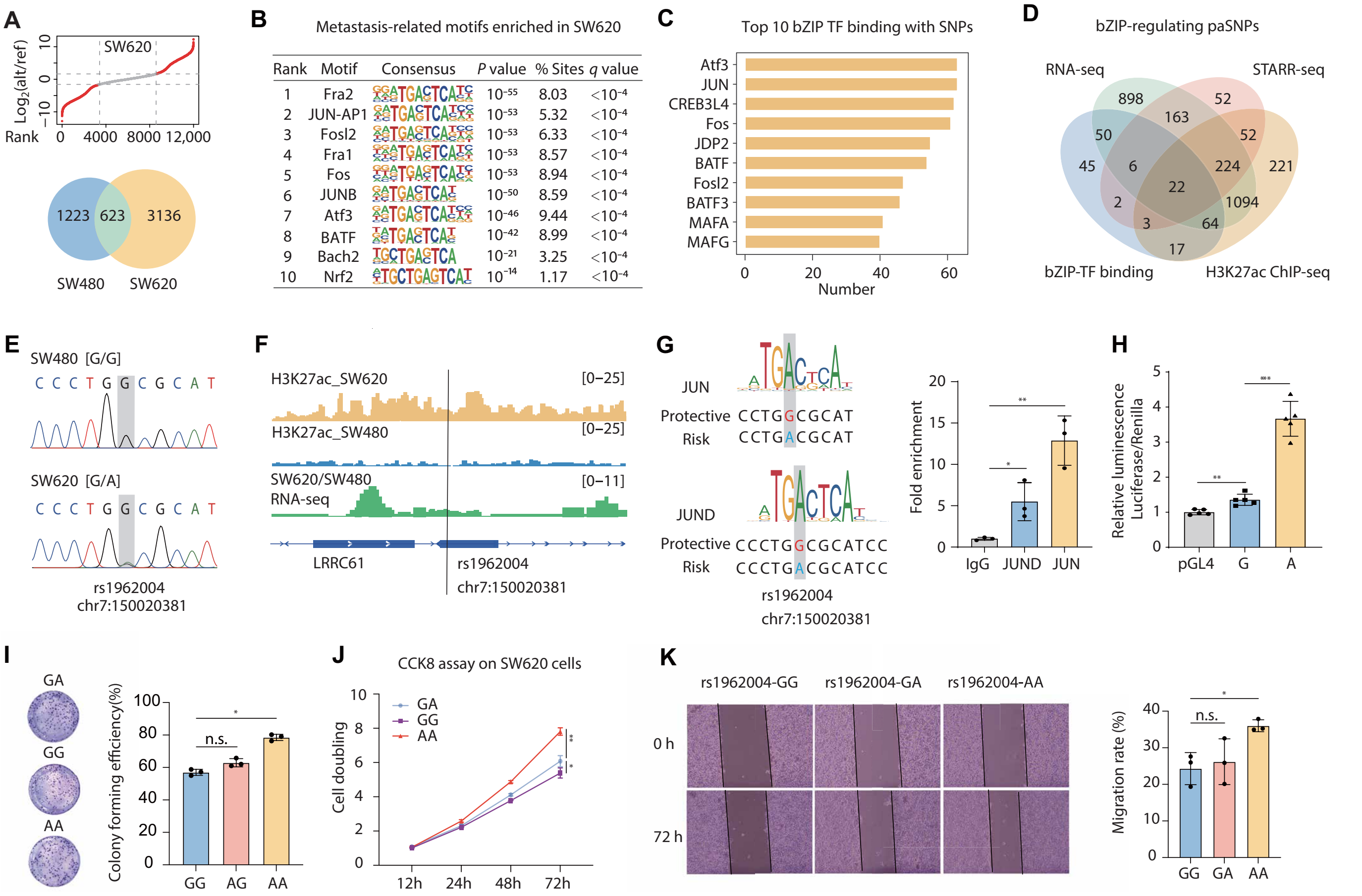

Fig. 4. Identification of a functional metastasis-related SNP rs1962004. (**A**) The roseplot diagram ranks the $\log_2$FC of alt/ref EAS and selects the significant paSNPs in SW620 (cutoff: left, −1.58; and right, 1.64). The Venn diagram illustrates the preferentially active loci in SW480 and SW620 cells, highlighting 3136 metastasis-specific loci unique to SW620. (**B**) Analysis predicted enrichment of bZIP family TFs at these SNPs, which are primarily associated with CRC metastasis. (**C**) Bar chart shows the top 10 bZIP TFs ranked by the number of SNP binding sites. (**D**) Venn diagram illustrating functional metastatic loci identified by integrated multi-omics analysis of RNA-seq, H3K27ac ChIP-seq, and SNP-STARR-seq data in SW620 versus SW480 cell lines, with prioritized bZIP TF–regulated SNPs. (**E**) The Sanger sequencing to confirm the genotypes of SNP rs1962004 in SW480 (top) and SW620 (bottom) cells, respectively. (**F**) Visualization of the genomic location of rs1962004 along with H3K27ac ChIP-seq and RNA-seq epigenetic marks in SW480/SW620 cell lines using the IGV Browser. (**G**) The motif analysis indicated that rs1962004-G/A affects JUN and JUND binding. ChIP assay confirmed stronger JUN and JUND enrichment in SW620 cells. (**H**) Dual-luciferase reporter gene detection of enhancer activity differences in the 120-bp region containing rs1962004 G/A in SW620. The luciferase signal was normalized to Renilla activity. (**I**) Colony formation to detect the proliferation ability of SW620 with different genotypes (GG, GA, and AA) of rs1962004. (**J**) Proliferation ability of SW620 with different genotypes (GG, GA, and AA) of rs1962004 determined by CCK-8 assay. (**K**) Wound healing to evaluate the migration of SW620 with different genotypes of rs1962004 (GG, GA, and AA) at 0 and 72 hours (h). $*P < 0.05$, $**P < 0.01$, and $***P < 0.001$. n.s., not significant.

H3K27ac ChIP-seq peaks, we demonstrated that 1697 of these SNPs were situated in metastasis-activated enhancers. Collectively, these results underscore that SNP-STARR-seq is an effective tool for identifying functional genetic variants in a cell-type–specific manner, facilitating the discovery of CRC metastasis–associated risk SNPs.

The role of SNP rs1962004 in driving expression of *LRRC61*

Given the pronounced enrichment of bZIP-family TF-binding motifs at these loci, we hypothesized that SNPs disrupting bZIP binding drove enhancer hijacking during metastatic progression. We therefore prioritized 22 high-confidence metastasis-associated SNPs (Fig. 4D and table S6) on the basis of four criteria, including: (i) significant allele-specific enhancer activity in SW620 (but negative in SW480), (ii) being located within SW620-specific H3K27ac peaks, (iii) in proximity (±1 Mbp) to metastasis–up-regulated genes, and (iv) capable of disrupting the putative bZIP-family TF-binding sites. Among these prioritized variants, we found that one SNP rs1962004 displayed different genotypes between SW480 and SW620 cells (SW480, G/G; and SW620, G/A; Fig. 4E), indicating that the SW620-specific "A" allele was highly likely a somatically obtained mutation and could contribute to the metastasis of the CRC cells. Thereby, we selected the SNP for subsequent mechanistic characterization. In comparison to SW480 cells, the SW620 cells showed acquired H3K27ac enrichment at this locus, revealing the enhancer activity of the neighborhood. Meanwhile, we observed a significantly stronger expression of adjacent gene *LRRC61* in SW620 cells compared to that in SW480 cells, indicating the potential regulatory relationship between the metastasis-specific enhancer and *LRRC61* gene expression (Fig. 4F).

As discussed above, noncoding mutations usually led to altered TF binding and aberrant expression of target gene. SNP rs1962004 was predicted to reside within a putative binding site of bZIP TFs JUN and JUND. Hence, we first confirmed the significant binding of both JUN and JUND to this locus using ChIP-qPCR (Fig. 4G). The functional redundancy aligned with established mechanisms of bZIP TFs, where JUN and JUND frequently form heterodimers as part of the AP-1 complex to cooperatively regulate target genes involved in oncogenesis and metastasis (*26*–*28*). Notably, these TFs preferred the "A" allele acquired by SW620, suggesting that a new binding site of JUN and JUND was created at the locus during metastasis when SW620 underwent "G"-to-"A" transition of rs1962004. This was also consistent with the observed epigenetic changes in the surrounding locus from SW480 metastasized to SW620 cells (Fig. 4F). Furthermore, a luciferase reporter assay demonstrated significantly higher activity of the genomic fragment containing the "A" allele compared to the "G" allele ($P < 0.001$, Fig. 4H).

Additionally, by using CRISPR-Cas9–based genome editing, we generated two cell clones with homozygous SNP rs1962004 genotypes: G/G and A/A, respectively. Our results consistently showed that cells with the A/A genotype displayed significantly greater proliferative and migratory abilities compared to SW620 cells with heterozygous rs1962004 (i.e., G/A genotype), which, in turn, exhibited greater prometastatic abilities than homozygous G/G cells (Fig. 4, I to K). Complementarily, deletion of the 104-bp enhancer region spanning rs1962004 (fig. S3, D and E) significantly attenuated proliferation in SW620 cells. Meanwhile, *LRRC61* expression was significantly down-regulated in enhancer-KO cells compared to that in wild-type controls ($P < 0.01$, fig. S3F). These findings altogether demonstrate that both the acquired "A" allele and the enhancer element itself mechanistically drive metastatic aggressiveness. Binding of JUN or JUND, caused by "G"-to-"A" transition at rs1962004, consequently enhanced cis-regulatory activity and up-regulated expression of *LRRC61*.

The metastatic role of *LRRC61*

To further establish the regulatory relationship between the SNP and *LRRC61*, we referred to eQTL data from the TCGA-CRC cohort and revealed that *LRRC61* exhibited correlated expression with rs1962004 genotypes ($P = 7.19 \times 10^{-19}$). Namely, individuals carrying the A/A genotype had significantly higher *LRRC61* expression, followed by A/G and G/G carriers (Fig. 5A). In concert with the broader phenotypic trends observed at the population level, edited SW620 cells with an A/A genotype also showed elevated *LRRC61* levels compared to cells of the same origin carrying the G/G genotype (Fig. 5B). Previous analysis revealed metastasis-related JUN and JUND enrichment at the rs1962004 locus. Here, we performed siRNA-mediated knockdown of JUN and JUND, both of which attenuated *LRRC61* expression (Fig. 5, C and D).

The leucine-rich repeat containing (LRRC) protein superfamily has complex and diverse functions in various solid tumors (such as sarcoma, melanoma, and glioblastoma), with some members (such as *LRRC15* and *LRRC32*) driving tumor invasion and metastasis by regulating transforming growth factor–β signaling, promoting cancer-associated fibroblasts activation, and influencing the immune microenvironment (*29*, *30*). However, the role of *LRRC61* in cancer, especially CRC, had not been well understood. Here, we intended to investigate its potential involvement in driving metastatic phenotypes. Knockdown of LRRC61 ($P < 0.001$) using short hairpin RNA (shRNA) led to decreased clonogenicity and migration (Fig. 5, E to G), which were phenotypes closely associated with metastasis. Metastasis significantly causes patient mortality and poor prognosis. Clinical follow-up of the patients with CRC revealed that *LRRC61* expression was higher in tumor tissues compared to that in normal tissues (Fig. 5H), and patients with elevated *LRRC61* expression generally had a poorer prognosis (Fig. 5I). A genetic model for this regulation proposes that the risk allele rs1962004-A, by enhancing JUN/JUND binding and up-regulating *LRRC61* expression, underlies the increased metastatic potential and poorer patient prognosis (Fig. 5J).

Methyl-STARR-seq identifies methylation-dependent enhancer elements

Alongside genetic variants that influence disease phenotypes, epigenetic variations are also widely acknowledged as key etiological factors affecting human health. DNA methylation represents a critical epigenetic mechanism regulating gene expression. While existing research predominantly focuses on promoter methylation, the regulatory roles and precise genomic mapping of methylation within enhancer regions, which constitute a substantial proportion of the epigenome, remain inadequately characterized. To address this gap, we designed an oligo library targeting differentially methylated enhancer regions in CRC and 120-nt windows centered at H3K27ac peak summits. This library was synthesized and cloned into the CpG-free Methyl-STARR-seq vector (*14*). Following in vitro enzymatic methylation treatment with glycerol control or M.SssI, an enzyme that could catalyze complete methylation of CpG sequence uniquely in the insert fragments (*31*), we generated unmethylated and fully methylated version of the same plasmid pools. The conversion rate and methylation status were confirmed by bisulfite sequencing (BS-seq; fig. S4, A and B). These constructs

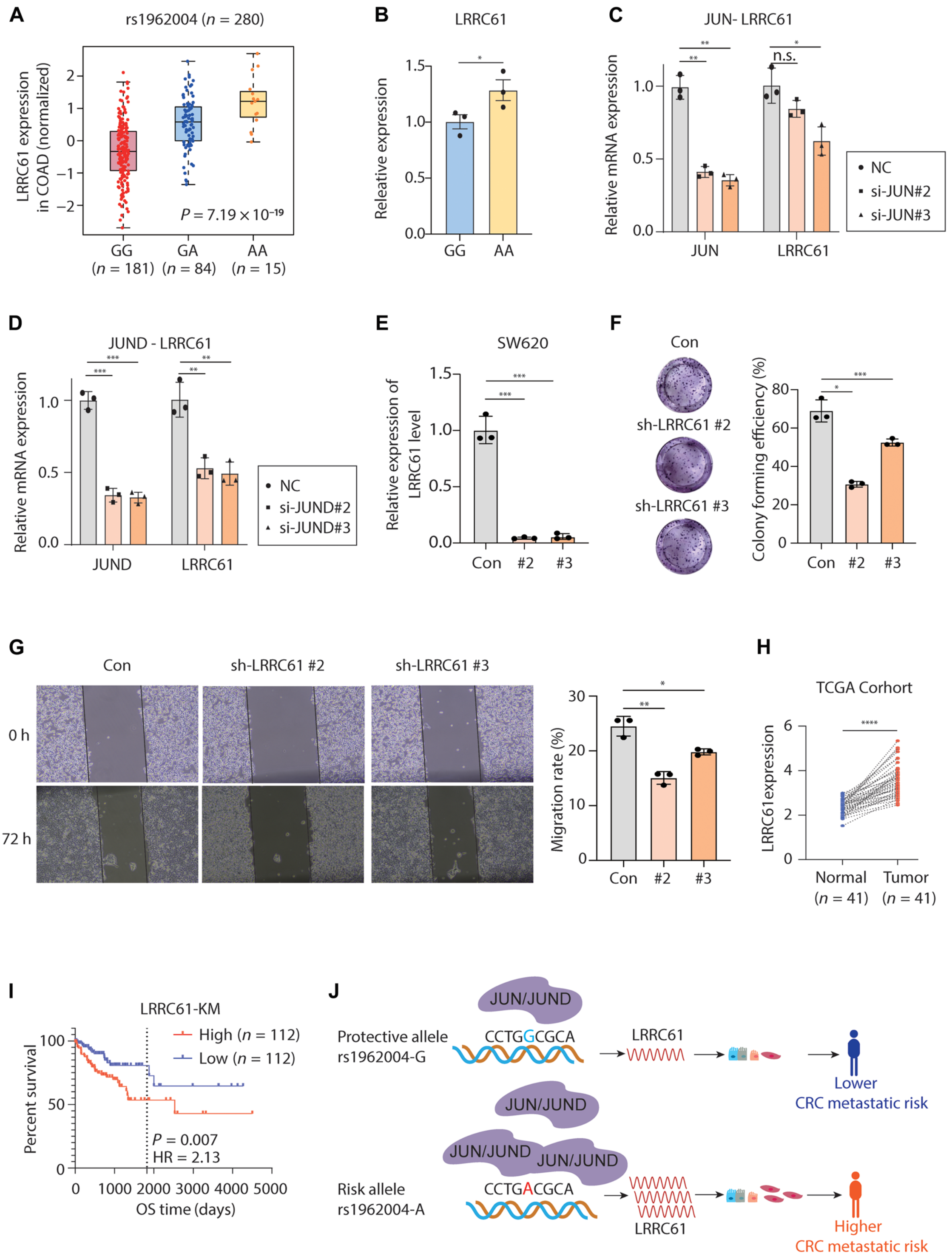

Fig. 5. rs1962004 regulates *LRRC61* expression. (**A**) The normalized expression levels of *LRRC61* in COAD samples ($n = 280$), stratified by rs1962004 genotype (GG, GA, and AA). Individuals with the AA genotype exhibit significantly higher *LRRC61* expression. The association was evaluated using a linear regression model implemented in the pancanQTL platform ($P = 7.19 \times 10^{-19}$). (**B**) Expression analysis of *LRRC61* in SW620 cells of rs1962004 homozygous cell lines (GG versus AA). (**C** and **D**) siRNA-mediated JUN or JUND knockdown suppressed *LRRC61*. (**E**) Knockdown efficiency of two LV-shRNA tested by qPCR. (**F**) Cell proliferation ability tested by colony formation assay. (**G**) Wound healing to evaluate the migration of SW620 of sh*LRRC61* at 0 and 72 hours (h). (**H**) Analysis of mRNA expression in paired TCGA-COAD samples revealed significant up-regulation of *LRRC61* in tumor tissues. (**I**) Kaplan-Meier survival analysis of patients with CRC stratified by *LRRC61* expression (high, upper quartile; and low, lower quartile). HR, hazard ratio; OS, overall survival. (**J**) Schematic model of rs1962004-mediated regulation of *LRRC61* in CRC metastasis. *$P < 0.05$, **$P < 0.01$, ***$P < 0.001$, and ****$P < 0.0001$. n.s., not significant.

were transfected into HCT116, SW480, and SW620 cell lines, respectively, to quantify their enhancer activity. Subsequently, mRNA and input DNA libraries were constructed and subjected to sequencing and bioinformatic analyses (experimental workflow outlined in Fig. 1). By calculating differential enhancer activity (EAS) between methylated and unmethylated states, we identified the most significantly influenced regulatory elements by DNA methylation in each cell line (HCT116, $n = 1763$; SW480, $n = 3588$; and SW620, $n = 4112$; fig. S4, C and D, and table S4).

Following methylation, ~60% of the tested fragments exhibited reduced enhancer activity in HCT116 cells, compared to ~40% in SW480 and SW620 cells, highlighting cell line-specific heterogeneity (Fig. 6A). For elements common to HCT116 and SW480, validation using a CpG-free Lucia reporter vector (InvivoGen) demonstrated high concordance with the high-throughput sequencing results (Fig. 6B and fig. S4E). The observed methylation-dependent alterations in enhancer activity likely stemmed from the methylation sensitivity of TFs. Analysis of TF-binding potential revealed predicted binding sites within more than 95% of these elements (table S8). Notably, nearly 80% of these sites contained CpG-rich motifs within their predicted TF-binding sequences, suggesting that CpG methylation might directly affect TF occupancy (Fig. 6C). Furthermore, the TFs enriched at these Methyl-STARR-seq–identified regulatory elements showed strong concordance with the established methylplus (defined as methylation of CpG within binding sites increased binding affinity) and methylminus (defined as methylation within binding sites of CpG decreased binding affinity) TF categories defined by Methylation Systematic Evolution of Ligands by Exponential Enrichment (Methyl-SELEX) (*32*), confirming the reliability of our screening system and also the role of TFs in modulating the methylation-dependent enhancer activity (Fig. 6D). We further performed a comprehensive HOMER motif enrichment analysis for the methylminus versus methylplus elements identified in HCT116, SW480, and SW620 cell lines, as well as specifically for the 3008 metastasis-acquired methylation-sensitive elements unique to SW620. Our results revealed that the methylminus elements in the more aggressive HCT116 and SW620 cell lines were preferentially enriched for ETS family TFs, well-established drivers of invasion and metastasis (*33*, *34*). In contrast, the primary SW480 line showed stronger enrichment for bZIP/AP-1 motifs. This notable context-specific pattern, particularly the strong ETS factor enrichment in metastasis-acquired, methylation-sensitive elements of SW620, provides a compelling mechanistic link between epigenetic reprogramming during metastatic progression and the activation of pro-metastatic transcriptional programs (fig. S5A).

We subsequently focused on DMRs potentially playing critical roles in CRC pathogenesis. Integrating TF motif analysis with H3K4me1 modification data, we identified six putatively functional CpG sites including cg25982657, cg08640619, cg23516310, cg14302214, cg14179401, and cg14106234, none of which had been previously characterized (Fig. 6E and table S9). Analysis of methylation data from paired tumor-normal samples across 14 cancer types revealed that cg25982657 and cg08640619 were specifically hypermethylated in CRC ($|\Delta\beta| > 0.25$, false discovery rate (FDR) < 0.05; fig. S5B and table S10].

Using cg08640619 as a representative site, we performed functional validation. This CpG site resided in the first intron of the *KIRREL1* gene that exhibited significant enhancer activity (Fig. 6F). In silico prediction indicated potential binding of RUNX2, a TF known to preferentially bind to unmethylated DNA. To confirm the role of DNA methylation on RUNX2 binding to this locus, we used CRISPR–dead Cas9 (dCas9)–mediated targeted methylation in HCT116 and SW480 cells. Subsequent ChIP-qPCR demonstrated significantly reduced enrichment of RUNX2 at the hypermethylated locus in HCT116 cells ($P < 0.001$, Fig. 6G). Knockdown of RUNX2 expression caused significant down-regulation of its adjacent genes *KIRREL1* and *ETV3* within the same TAD, confirming that RUNX2 bound preferentially to the unmethylated sequence and positively regulated *KIRREL1* and *ETV3* (Fig. 6H). Having observed a significant negative correlation between cg08640619 methylation and KIRREL1/ETV3 expression in clinical samples (Fig. 6I), we sought to functionally validate this finding. Using CRISPR-dCas9-DNMT3A–mediated targeted hypermethylation, we confirmed that increased methylation directly causes significant down-regulation of both genes, demonstrating a causal regulatory relationship (fig. S5C). *KIRREL1* expression was found to be significantly up-regulated in tumor tissues compared to matched normal tissues in TCGA–colon adenocarcinoma (COAD) paired samples (fig. S5D). This up-regulation may be associated with the hypomethylation status of cg08640619 in CRC tumors. Prognostic analysis of clinical data revealed that hypomethylation of cg08640619 is significantly associated with poor patient prognosis in advanced-stage disease (stages III to IV) (Fig. 6J).

Given the cancer-specific hypomethylation patterns (fig. S5B), we hypothesized that cg08640619 and cg25982657 could serve as sensitive biomarkers for the early detection of CRC. To evaluate the diagnostic potential of the identified CpG sites, we applied both logistic regression (LogR) and support vector machine (SVM) classifiers to samples across 14 different cancer types. In addition, a 10-fold cross-validation was performed to ensure model robustness (fig. S5, E, G, and H). Results showed that both CpG sites effectively discriminated CRC tumors from normal tissues, but not as well for other tumors (Fig. 6K and fig. S5F). Methyl-STARR-seq–detected functional epigenetic variants were highly correlated with disease risk, having great potential clinical values for specific and sensitive cancer diagnosis.

Methyl-STARR-seq identifies methylation-dependent metastasis

Similar to our screening for metastasis-associated functional SNPs, we identified 3008 methylation-sensitive regulatory regions specifically dysregulated in SW620 cells (Fig. 6L and table S11). Integrative analysis of H3K27ac modification profiles and RNA-seq data across both cell lines identified 182 genomic fragments exhibiting enhanced regulatory activity in the hypomethylated state (Fig. 6M). These loci concurrently showed significantly elevated H3K27ac enrichment in SW620 cells compared to that in SW480 cells and were located within ±1 Mb of genes significantly up-regulated in this metastatic line.

Take one such putative site, cg02205193, for illustration. These CpG dinucleotides are located near the promoter of *LINC00460*, a gene coding for a competing endogenous RNA that had been reported to promote CRC metastasis by a handful of studies (*35*–*37*). The genomic region enclosing cg02205193 exhibited markedly higher H3K27ac signals in SW620 cells compared to than in SW480 cells (Fig. 6N). Consistently, LINC00460 is significantly up-regulated in CRC metastatic patients as what we observed in the metastatic SW620 cell line, potentially attributable to its hypomethylation status (*38*). Furthermore, methylation at cg02205193 putatively influenced the binding of GFI1, a methylminus TF according to Methyl-SELEX data (*32*). Specifically, the hypomethylated state of cg02205193 facilitates enhanced GFI1 recruitment, consequently activating expression

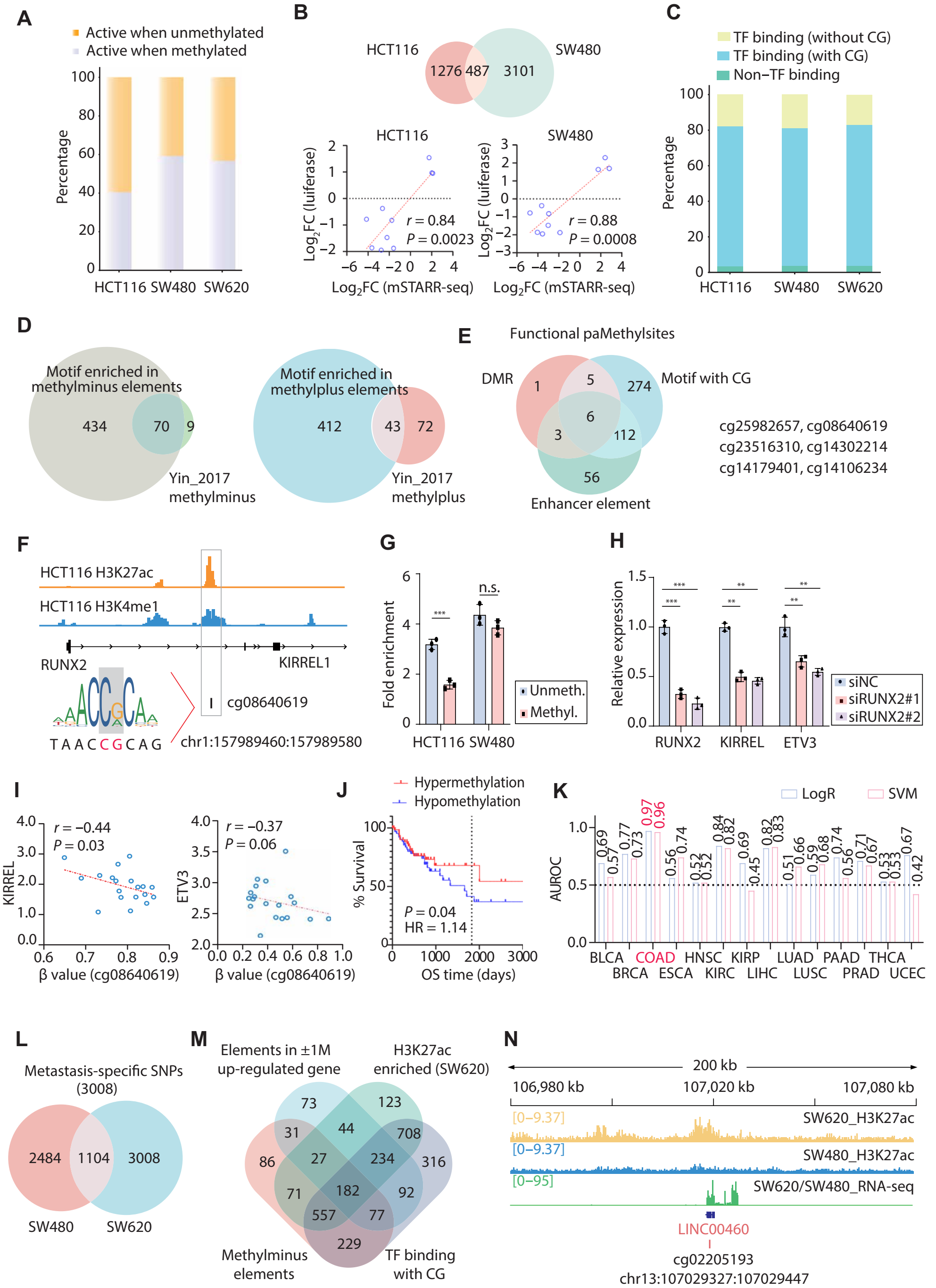

Fig. 6. Methyl-STARR-seq identifies methylation-dependent enhancer elements. (**A**) The bar chart displays the proportions of enhanced and diminished enhancer activity in three cell lines following methylation versus unmethylation treatment. (**B**) Overlap of methylation-sensitive loci in HCT116 and SW480 cells. CpG-free Lucia reporter assays validate Methyl-STARR-seq results for representative loci. *r*, correlation coefficient. (**C**) Proportion of TF motifs enriched at differential enhancer activity loci that contain CpG sequences. (**D**) Overlap between TFs enriched at methylation-sensitive loci and the methylplus/minus categories defined by Methyl-SELEX. (**E**) Integration of histone marks (H3K4me1 and H3K27ac), clinical differential methylation, and TF-binding data identifies six high-confidence functional CpG sites. (**F**) Genomic browser view of the cg08640619 locus within a *KIRREL1* intron, showing enhancer marks and a predicted RUNX2 binding site. (**G**) CRISPR-dCas9-DNMT3A–mediated targeted methylation at cg08640619 reduces RUNX2 enrichment (ChIP-qPCR) in HCT116 and SW480 cells. (**H**) The mRNA expression analysis of target genes *KIRREL1* and *ETV3* following RUNX2 knockdown. (**I**) Correlation between cg08640619 methylation (β value) and expression of *ETV3* and *KIRREL1* in clinical samples. (**J**) Kaplan-Meier analysis of overall survival based on cg08640619 methylation status. (**K**) Area under the receiver operating characteristic curve (AUROC) values for classifying cancer versus normal tissue across 14 cancer types using cg08640619 methylation, evaluated by logistic regression (LogR) and support vector machine (SVM). (**L**) Venn diagram depicting differentially significant methylation-sensitive loci in SW480 and SW620 cells, highlighting 3008 metastasis-specific regulatory elements unique to SW620. (**M**) Integration of RNA-seq, H3K27ac ChIP-seq, and Methyl-STARR-seq identifies functional, metastasis-associated methylation-sensitive loci. (**N**) Genomic browser view of the metastasis-associated locus cg02205193 and its target gene *LINC00460*, showing elevated H3K27ac signal in SW620 cells. ***P* < 0.01 and ****P* < 0.001. n.s., not significant.

of the target gene *LINC00460* and promoting CRC metastasis. On the basis of Hi-C and differential expression of genes between SW480/SW620 cells, we also identified regulatory regions enclosing previously uncharacterized CpG sites whose methylation status was associated with expression of *TCF12* and *HNF1* genes, two established genes implicated in CRC metastasis (fig. S5I) (*39*, *40*).

Thus, by integrating functional Methyl-STARR-seq screening with multi-omics validation, we could effectively identify potential diagnostic biomarkers. This approach not only delineates a precise regulatory landscape of epigenetic dysregulation in CRC but also provides a mechanistic foundation for identification of previously unidentified diagnostic and therapeutic biomarkers with broad clinical applicability.

DISCUSSION

In this study, we established an integrative functional genomics pipeline to systematically prioritize noncoding genetic and epigenetic variants driving CRC pathogenesis and metastasis. By combining SNP-STARR-seq (screening 30,790 SNPs) and Methyl-STARR-seq (profiling >134,000 CpG sites) in primary (HCT116 and SW480) and metastatic (SW620) CRC cells, we mapped allele- and methylation-dependent enhancer activities at unprecedented scale. We identified 1969 functional SNPs in HCT116 and 1846 in SW480 primary CRC cells, with 922 common variants driving enhancer dysregulation. Crucially, 3136 metastasis-acquired SNPs and 3008 methylation-sensitive elements were uncovered in SW620 metastatic cells. Mechanistic validation confirmed *TAF4* as a tumor suppressor regulated by rs67941642/rs6061231. We found a metastasis-acquired variant rs1962004 (A allele) enhancing *LRRC61* expression via JUN/JUND binding, promoting CRC progression and correlating with poor prognosis. Furthermore, we identified hypermethylated loci, like cg08640619 and cg25982657, as high-performance diagnostic biomarkers (AUROC > 0.96). This integrative approach bridges both genetic and epigenetic variation to transcriptional reprogramming in CRC pathogenesis and metastasis.

Our study, alongside recent work in primary normal cells (*10*), highlights that the functional readout of noncoding variants is profoundly shaped by cellular context. The enhanced regulatory activity that we observed in CRC cell lines likely reflects the aberrant transcriptional and epigenetic environment of advanced tumors, which unveils a broader spectrum of functionally relevant variants. This context specificity, coupled with our isogenic metastatic model, was critical for pinpointing regulatory drivers of metastasis. The functional annotation of noncoding SNPs in cancer has been revolutionized by the key technologies, MPRA or STARR-seq. MPRA, pioneered as a "regulatory lie detector," enables simultaneous testing of thousands of DNA fragments for enhancer activity by coupling candidate sequences to barcoded reporters (*41*, *42*). While these methods have successfully linked GWAS risk loci to regulatory mechanisms in various contexts (*42*–*46*), their application in CRC, particularly concerning metastasis, has remained limited and constrained by specific design choices. For instance, the most comprehensive prior CRC SNP study used MPRA but focused exclusively on variants within statistically fine-mapped GWAS risk loci, testing only 8880 variants in primary CRC cells (*46*). This approach, while valuable, inherently overlooked the vast landscape of functional regulatory SNPs residing outside GWAS-implicated regions and the critical context of metastatic progression. Our study fundamentally advances beyond these limitations through a conceptually distinct and more expansive strategy: (i) Dual-scale variant screening: We implemented a dual-scale SNP-STARR-seq screening strategy. Unlike previous studies prioritizing only GWAS-linked variants, we systematically interrogated both GWAS-associated SNPs (6363) and enhancer-localized SNPs independent of GWAS hits (24,526), totaling 30,790 unique variants. This unbiased approach significantly broadened the scope beyond known risk loci, capturing a more comprehensive picture of functional noncoding variation within CRC enhancers. (ii) Incorporating the metastatic context: Crucially, we performed screening not only in primary CRC cell lines (HCT116 and SW480) but also in the isogenically paired metastatic line SW620, derived from the same patient's lymph node metastasis. This unique paired model is pivotal, as it allowed us to directly compare enhancer activity landscapes and pinpoint 3136 metastasis-acquired regulatory SNPs specifically functional in the metastatic niche, a dimension entirely absent in previous large-scale CRC functional genomics studies. The functional characterization of these metastasis-specific variants revealed a notable and significant enrichment of binding motifs for bZIP-family TFs (e.g., JUN and JUND). This finding strongly implicates the dysregulation of bZIP/AP-1 complexes, the master regulators of epithelial-mesenchymal transition and metastasis (*16*, *22*–*25*), as a key consequence of somatic and regulatory evolution during CRC metastatic spread. The identification of this specific TF family enrichment among metastasis-acquired variants is an original insight uniquely enabled by our metastatic context screening.

DNA methylation is well-established as a crucial epigenetic mechanism in cancer diagnosis (*47*–*49*). However, existing research on cancer methylation has primarily focused on promoter regions, overlooking the significant potential of enhancer regions, which account for a substantial proportion of the epigenome (*9*). In this study, we used Methyl-STARR-seq (*14*), which was less extensively applied in cancer context, to systematically investigate enhancer region methylation in CRC. Crucially, because both SNPs and methylation sites were selected from identical enhancer regions, this integrated approach provides mechanistic insights into the regulatory architecture of CRC enhancers, delineating genomic segments influenced by genetic variation versus those preferentially modulated by epigenetic alterations (table S1). By integrating Methyl-STARR-seq approach with machine learning classifiers, we identified two potential early-warning biomarkers, cg08640619 and cg25982657. These two differentially methylated probes represent CRC-specific methylation variations that exhibit remarkable diagnostic potential (Fig. 6K, fig. S5F, and table S10).

Our integrated functional genomics framework establishes a blueprint for deciphering noncoding drivers across diverse malignancies. The methodology and insights derived from this CRC-focused study offer key translational implications for other cancer types: pan-cancer applicability of the screening pipeline. The dual SNP-/Methyl-STARR-seq platform, coupled with multi-omics characterization, is readily adaptable to other epithelial cancers. For example, enhancer-centric variant selection (prioritizing H3K27ac/ATAC-defined regions) bypasses GWAS limitations in understudied cancers (e.g., pancreatic adenocarcinoma), enabling de novo discovery of regulatory drivers. In vitro methylation manipulation via M.SssI permits systematic profiling of methylation-sensitive enhancers in cancers with prominent epigenetic dysregulation [e.g., glioblastoma isocitrate dehydrogenase (IDH)–mutant subtypes].

Predictive and diagnostic biomarker development: Our biomarker discovery strategy, from functional screening to causal SNP or enhancer differentially methylated regions (eDMR) detection, provides

a template for other malignancies: Tissue-specific hypermethylated enhancers (e.g., *cg08640619* in our study) could be exploited in cancers lacking sensitive biomarkers (e.g., early-stage pancreatic cancer). The exceptional diagnostic performance (AUROC > 0.96) achieved by our CRC-specific enhancer methylation markers, cg08640619 and cg25982657, significantly surpasses the typical performance range reported for promoter-based methylation biomarkers in CRC early detection, which often exhibit AUROC values between 0.80 and 0.90 (*50–52*). This substantial improvement underscores the power of functionally screening epigenetic variants within enhancer elements, rather than relying solely on promoter-centric profiling. These functionally informed epigenomic signatures not only provide superior diagnostic accuracy but also pinpoint regulatory elements mechanistically linked to disease pathogenesis.

While our integrated approach provides a comprehensive functional annotation of noncoding variants in CRC, several limitations warrant consideration. Regarding library design constraints imposed by oligonucleotide synthesis limitations, the genomic fragments captured (excluding adapter sequences) were restricted to 120-nt windows. This 120-bp scale may be insufficient to fully recapitulate long-range chromatin interactions or topological domain effects. Furthermore, for methylation profiling, while the 18,880 enhancer regions harbored over 950,000 CpG sites, substantial variation in enhancer lengths (ranging from hundreds to thousands of base pairs) necessitated focusing our synthesized library on 120-nt segments centered at H3K27ac peak summits. We acknowledge that our targeted design necessarily misses certain classes of functional loci: latent or poised enhancers: regions that are not marked by H3K27ac in the cell lines we profiled (e.g., weak enhancers and enhancers active in other CRC subtypes or under specific stimuli) would be absent from our library. Furthermore, our cellular models, while providing controlled experimental conditions, lack the complexity of tumor microenvironments (e.g., immune-stromal interactions) that may modulate enhancer activity in vivo. Future studies should prioritize: (i) in situ fragmentation strategies to construct size-diverse libraries encompassing full enhancer architectures; (ii) single-cell multi-omics approaches to dissect allele- and methylation-specific regulatory heterogeneity during metastatic progression; and (iii) while we have validated the diagnostic potential of cg08640619 and cg25982657 in tissue samples, their application as liquid biopsy biomarker requires further investigation and development. Addressing these dimensions will bridge the gap between enhancer maps and actionable therapeutic contexts.

MATERIALS AND METHODS

Oligo design

We established a comprehensive SNP set through integrated functional and disease-relevance criteria: (i) applied stringent MAF filtering [≥0.05 across AMR/EUR/EAS/SAS in 1000 Genomes Project (*15*)]; (ii) expanded 238 CRC GWAS variants via LD-proxy imputation ($R^2 \geq 0.5$, ±500 kb) yielding 6125 additional SNPs; and (iii) independently prioritized 24,526 enhancer-localized SNPs defined by chromatin coaccessibility [intersection of H3K27ac ChIP-seq (ENCSR661KMA/ENCSR000EUT) and ATAC-seq peaks (ENCSR872WGW) in HCT-116 cells]. The union set comprised 30,790 unique SNPs (6363 GWAS-derived and 24,526 enhancer-based, with 99 overlaps).

For methylation-specific oligos, we selected (i) the enhancer-core CpGs: more than 134,000 CpG dinucleotides within ±60 bp of H3K27ac peak summits; and (ii) CRC-differential CpGs ($|\Delta\beta| > 0.3$, FDR < 0.05): 908 enhancer-localized probes from Illumina 450K arrays from TCGA-CRC cohort, GSE139404, GSE77955, GSE53051, GSE48684, GSE42752, and GSE40055. Oligonucleotide design was adapted to a Beijing Genomics Institute Sequencing (BGI) system, and the two libraries were synthesized separately by CustomArray. In brief, each oligonucleotide contains 120 bp of genomic sequence enclosing the SNP or CpG sites and constant flanking sequences (upstream, 5′-ATGGCTACGATCCGACTT; and downstream, AAGTCGGAGGCCAAGCGGTCTTAGGAAGACAA-3′) on both ends, which were used for cloning.

SNP-STARR-seq plasmid preparation

The hORI-STARR-seq vector was provided by A. Stark (Research Institute of Molecular Pathology, Vienna, Austria). The PCR-amplified SNP library (20 ng) was inserted into Age I/Sal I sites of the plasmid (100 ng) using highly efficient recombination-based cloning (ClonExpress, Vazyme, China). To avoid biases during the cloning, we performed a total of 20 separate recombination reactions and pooled every four reactions. After 1× AMPure XP DNA bead purification, we used the SNP-containing vectors (2 μl) to transform extra-competent *Escherichia coli* DH5α (DH5α; 50 μl). Again, we performed 20 separate transformation reactions and pooled every four transformations. The plasmids were then extracted and purified at a high concentration (>1 μg/μl). PCR amplification from the plasmids was performed and sequenced for 2 × 100 paired-end cycles as input control.

Methyl-STARR-seq plasmid preparation

The pmSTARRseq1 was a gift from J. Tung. The methods for cloning are similar to those of the aforementioned SNP-STARR-seq, except that the PCR-amplified methylation library (10 ng) was inserted into Spe I/Nco I sites of the plasmid (100 ng). The plasmids were transformed into extra-competent GT115 cells (with Zeocin screening at 50 μg/ml). We then methylate half of the plasmid library with M.SssI [DNA, 4 U/μg; New England Biolabs (NEB)] and mock treat the other half (using the same reaction protocol, but replacing the enzyme with ddH_2O). To confirm the DNA methylation status of plasmids, we first digested the plasmid DNA (1 μg) by incubation (at 37°C) with Xba I and Nhe I with a total of five reactions. Approximately 1 ng of unmethylated enhanced green fluorescent protein fragment was spiked in with each sample for the assessment of the bisulfite conversion efficiency. We performed end repair, A-tailing, and adapter ligation. The samples were then subjected to sodium bisulfite conversion using an EZ DNA Methylation-Gold kit (Zymo Research). Libraries were then PCR amplified (KAPA HiFi Uracil+ DNA Polymerase) and sequenced on the Illumina platform.

Cell culture and transfection

Each oligo pool was transfected into human embryonic kidney (HEK) 293T or CRC cell lines (HCT116, SW480, and SW620) using jetOPTIMUS (Polyplus). The cells were cultured under normal condition with 5% CO_2 at 37°C. Specifically, 2 μg of plasmids were mixed with 3 μl of transfection reagents for transfection into cells cultured in a single well of six-well plate. We pooled every six wells (~2×10^7 cells) for one biological repeat.

mRNA extraction and sequencing

Thirty-six hours posttransfection, total RNA was then extracted with an RNeasy kit (TAKARA), and mRNA was enriched with Poly(A) mRNA Magnetic Isolation Module (NEB, E7490L). First-strand cDNA

was synthesized using a specific primer (5′-CTCATCAATGTATCTTATCATGTCTG for SNP-STARR-seq and 5′-CAAACTCATCAATGTATCTTATCATG for methyl-STARR-seq) with SMARTScribe Reverse Transcriptase (Takara Bio, 639538). We amplified the cDNA obtained from reverse transcription for BGI sequencing using a two-step nested PCR. For the junction PCR (15 cycles), the forward primer spans the splice junction of the synthetic intron and specifically amplifies the reporter cDNA without amplifying any plasmid DNA (see primers in table S12). The entire purified products served as the template for the Index-PCR (10 cycles). DNA was purified with AMPure beads and sequenced for 2 × 100 paired-end cycles with BGI sequencer. In total, three biological replicates were performed with three technical replicates each for all cell lines.

Dual-luciferase reporter assay

To investigate the functional impact of SNPs or methylation sites on enhancer activity, dual-luciferase reporter assays were performed. Briefly, 120-bp DNA fragments harboring distinct SNP alleles or CpG-containing fragments were cloned into the pGL4.23 luciferase reporter vector (Kpn I/Xho I sites) or the pCpG-free-lucia vector (BamH I/Spe I *sites*), respectively. The pGL4.23 or pCpG-free-lucia recombinant plasmid (firefly luciferase reporter) was cotransfected with the pGL4.74 vector to normalize for variations in transfection efficiency and cell viability. After 48 hours of transfection, luciferase activity was measured using the Dual-Lumi Luciferase Assay Kit (Beyotime, RG088S). Relative enhancer activity = Firefly luminescence/Renilla luminescence. The cloning primers as well as the sequences of detected variant sites are listed in table S13. The original data for Firefly and Renilla luminescence were listed in table S14.

RNA sequencing

Total RNA was extracted from cells using the TAKARA MiniBEST Universal RNA Extraction Kit. Polyadenylated RNA was enriched from 5 μg of total RNA, followed by direct elution using a preformulated reverse transcription system for cDNA synthesis. Residual RNA was removed by ribonuclease (RNase) A/H treatment. The cDNA was purified and recovered using AMPure XP beads, and adapters were ligated at the 5′ end. After two rounds of purification with AMPure XP beads, the DNA was amplified using BGI sequencing primers (BGI-system F and BGI-Barcode R), with distinct downstream primers used for different samples to enable sample multiplexing. Following amplification, the DNA was further enriched using AMPure XP beads before pooling for sequencing.

Genotyping

Genomic DNA was extracted from cells following the manufacturer's protocol of the DNA extraction kit. Primer pairs flanking the target SNP locus were designed (table S15) and used for PCR amplification with Taq polymerase. The PCR products were directly submitted to a biotechnology company for Sanger sequencing. The genotype of the SNP locus was determined by analyzing the sequencing chromatogram peaks at the target position (SnapGene).

CRISPR-Cas9–mediated gene editing

Knockout

To elucidate the functional role of specific SNP-containing regions as potential enhancers, we used CRISPR-Cas9–mediated gene editing to generate targeted KO of the SNP site fragments. This approach enabled the assessment of enhancer activity through quantitative analysis of downstream gene expression changes.

Two single guide RNAs (sgRNAs) flanking the target SNP region were designed using the CRISPR design platform CHOPCHOP (https://chopchop.cbu.uib.no/). sgRNAs were selected on the basis of stringent criteria, including high on-target efficiency (≥60%) and minimal off-target effects, as predicted by the tool. The selected sgRNAs were subsequently cloned into the pX333 vector, which coexpresses Cas9 and the sgRNAs for efficient genome editing. The plasmid was transfected into HCT116 cells using optimized transfection protocols. After 24 hours, the medium was replaced, and cells were subjected to selection with puromycin (1.5 μg/ml). Selection was maintained until near-complete cell death was observed in the nontransfected control group. Transfected cells were then harvested, and ~500 cells were seeded into a 10-cm dish to facilitate clonal expansion.

Cells were cultured for 10 days to allow the formation of monoclonal colonies. Colonies reaching a diameter of 1 mm were manually isolated and transferred into 48-well plates for further expansion. Upon reaching confluence, genomic DNA was extracted from each clone, and the target region was amplified by PCR. Successful KO of the SNP-containing fragment was confirmed by Sanger sequencing, which validated the precise deletion of the target region.

Point mutation

To precisely delineate the functional consequences of SNPs on enhancer activity, we implemented a CRISPR-Cas9–based homology-directed repair (HDR) strategy to introduce specific point mutations at SNP sites. This approach enabled the generation of isogenic cell lines with defined genetic alterations, providing a robust platform for investigating the role of SNPs in gene regulatory mechanisms. sgRNAs targeting the SNP sites were designed using the CHOPCHOP online tool (https://chopchop.cbu.uib.no/) and cloned into the pSpCas9(BB)-2A-Puro (PX459) V2.0 vector (Addgene, no. 62988). This vector coexpresses Cas9, and the sgRNA and confers puromycin resistance for efficient selection of transfected cells. A 99-nt single-stranded oligodeoxynucleotide (ssODN) was synthesized in vitro to serve as a repair template for HDR. The ssODN was designed to incorporate the desired point mutation while introducing silent mutations at the protospacer-adjacent motif site to prevent recleavage by Cas9. This design ensured precise editing and minimized unintended genomic alterations. The pX459 plasmid (500 ng) and ssODN donor (20 μM) were cotransfected into 116 cells at 12-well plates using JetPRIME protocols. After 24 hours, the medium was replaced, and cells were subjected to selection with puromycin (1.5 μg/ml). Selection was maintained until near-complete cell death was observed in the nontransfected control group, ensuring enrichment for successfully transfected cells. Selection and cloning followed the KO protocol. All sgRNA sequences are listed in table S16.

CRISPR-dCas9–mediated targeted DNA methylation

Targeted DNA methylation of the cg08640619 locus was achieved using a CRISPR-dCas9-DNMT3A system. Briefly, guide RNA (sgRNA) designed to target the genomic sequence surrounding cg08640619 was cloned to a plasmid expressing a catalytically dCas9 fused to the catalytic domain of DNMT3A. A nontargeting sgRNA was used as a negative control. HCT116 cells were harvested 72 hours post-transfection. The impact on gene expression was assessed by reverse transcription (RT)–qPCR analysis of the putative target genes *KIRREL1* and *ETV3*.

CCK-8 assay

Cells from both control and experimental groups were seeded in 96-well plates at a density of 1×10^4 cells per well, with four replicate

wells for each group. At the designated time points (0, 24, 48, and 72 hours), Cell Counting Kit-8 (CCK-8) reagent [UElandy (UE), 10 μl per well] was added to each well. After 1.5 hours of incubation at 37°C, the absorbance at 450 nm was measured using a microplate reader (BioTek). The relative proliferation rate was calculated by normalizing the OD_{450} (optical density at 450 nm) values of experimental groups to those of the control group at 12 hours (Relative proliferation rate = OD_{450} experimental group/OD_{450} control group).

Colony formation assay
Cells (500 per well) were seeded in six-well plates. After 14 days, colonies were fixed [4% paraformaldehyde (PFA)], stained (0.1% crystal violet), and counted (ImageJ; colonies of >50 cells). The experiment was performed in triplicate.

Wound-healing assay
The wound-healing assay is a simple and widely used method for evaluating cell migration capacity by creating an artificial scratch in a confluent cell monolayer and monitoring the migration of cells to close the wound. Cells were cultured in six-well plates until reaching 90% confluence, followed by the creation of uniform linear scratches using a 10-μl pipette tip. After phosphate-buffered saline (PBS) washing, the medium was replaced with fresh medium containing 2% fetal bovine serum (FBS). Wound closure was documented at 0 and 72 hours using a Nikon Eclipse Ti microscope. The migration rate was quantitatively analyzed with ImageJ software and calculated as: Migration rate (%) = [(Initial area − Final area)/Initial area] × 100%.

Transwell assay
The Transwell assay is used to investigate cell migration and invasion capabilities. Cells were cultured to 80 to 90% confluence and serum-starved for 8 hours before trypsinization. Detached cells were resuspended in serum-free medium, and 1×10^5 cells in suspension were carefully loaded into the upper chamber. The lower chamber was filled with medium containing 10% FBS as a chemoattractant. Following 48-hour incubation at 37°C with 5% CO_2, nonmigratory cells and residual Matrigel were removed from the upper chamber using cotton swabs. The membrane was washed twice with ice-cold PBS, fixed with 0.4% paraformaldehyde for 10 min, and stained with 0.1% crystal violet for 10 min. Invaded cells were quantified by microscopic examination and image analysis software.

siRNA and shRNA knockdown
Gene silencing was performed using siRNA oligonucleotides targeting genes of interest. Cells were seeded in six-well plates at 2.5×10^5 cells per well and cultured for 24 hours to reach 40 to 50% confluence. Transfection complexes were prepared by incubating 30 nM siRNA (final concentration) with 4 μl of JetPrime transfection reagent (Polyplus) in 200 μl of serum-free medium for 10 min at room temperature. Complexes were added dropwise to cells in complete medium. After 48 hours of incubation, cells were harvested for functional assays or RNA extraction.

The shRNA sequences were designed and cloned into the pLKO.1 vector (Addgene, no. 10878). Lentiviral particles were packaged in HEK293T cells using psPAX2 and pMD2.G plasmids. Cells were infected with the packaged lentiviruses and selected with puromycin (2 μg/ml) for 72 hours. Knockdown efficiency (>70% mRNA reduction) was validated by RT-qPCR. All siRNA and shRNA sequences are listed in table S17.

Chromatin immunoprecipitation
ChIP assays were carried out as previously described. Briefly, the cells were fixed with 1% formaldehyde (Sigma-Aldrich, F8775) for 10 min at room temperature, and a final concentration of 125 mM glycine was used to quench the reaction. Cells were washed twice with cold PBS and collected in cold PBS by scraping and then resuspended in hypotonic lysis buffer [20 mM Hepes (pH 7.9) with 10 mM KCl, 10% glycerol, 1 mM dithiothreitol, and a protease inhibitor cocktail (04693124001, Roche)]. The pellet of nuclei was resuspended in radioimmunoprecipitation assay (RIPA) buffer [10 mM tris-HCl (pH 8.0) with 140 mM NaCl, 1% Triton X-100, 1 mM EDTA, 0.1% SDS, 0.1% sodium deoxycholate, and protease inhibitor]. Nuclear extracts were sonicated to generate chromatin fragment on a Covaris M220 Focused-ultrasonicator (insert, microTUBE 130 μl; temperature, 7°C; peak incident power, 75 W; duty factor, 5%; treatment time, 300 s). The chromatin was cleaned by centrifugation at 14,000*g* for 10 min at 4°C and diluted with 1 ml of RIPA buffer per reaction. Then, the chromatin was precleared with Protein G Sepharose 4 Fast Flow (17061801, GE) for 2 hours, and 30 μl of sample was set aside as an input and subjected to immunoprecipitation with antibody overnight. On the following day, Pierce Protein A/G Plus Agarose beads (Thermo Fisher Scientific, 20423) that were blocked with RIPA containing 0.5% bovine serum albumin were added into the samples followed by incubation for 2 hours in cold room under rocking. The beads were then washed three times with RIPA buffer, followed by two times in RIPA buffer with 0.5 M NaCl, once in LiCl buffer [250 mM LiCl, 1 mM EDTA, 0.5% IGEPAL CA-630, 0.1% sodium deoxycholate, and 10 mM tris-HCl (pH 8.0)], and twice in ice-cold tris-EDTA (TE) buffer. Each time, the beads were sustaining for 5 min with a gentle rock. After washing, both the beads and input sample were added with 150 μl of extraction buffer (1% SDS in 1× TE, 12 μl of 5 M NaCl, and 10 μg of RNase A) and incubated at 37°C for 1 hour with shaking. The DNA fragments were purified using DNA purification spin columns for subsequent qPCR with primers listed in table S18.

Library preparation for heterozygous SNPs involved a two-step PCR protocol. The first-round PCR amplified target fragments flanking the SNP locus, incorporating universal sequencing adapters. The second-round PCR added sample-specific index using the first-round PCR amplicons as template. Library preparation for H3K27ac ChIP-seq was performed using the NEBNext DNA Prep Kit (NEB, E7645). The library was sequenced using the DNBSEQ-T7 PE150 platform (Biopharmaceutical Public Service Platform, Nanjing)

Real-time qPCR
Total RNA was extracted from cells using TRIzol reagent and quantified. Genomic DNA was removed by treating 1.5 μg of RNA with 4 μl of 4× gDNA wiper Mix in a 200-μl PCR tube, adjusted to a final volume of 16 μl with RNase-free H_2O. The mixture was thoroughly mixed by pipetting and incubated at 42°C for 2 min. Reverse transcription was performed by adding 4 μl of 5 × HiScript II qRT SuperMix II, followed by mixing and incubation at 50°C for 15 min and 85°C for 5 s. The resulting cDNA was diluted 20-fold with H_2O and mixed well for immediate use in qPCR. Glyceraldehyde-3-phosphate dehydrogenase served as the internal reference gene, and relative gene expression was calculated using the $2^{-\Delta\Delta Ct}$ method. All qPCR primers are listed in table S17.

Analytic methods
Enhancer definition and pan-cancer enhancer map construction
We selected representative cell lines from 20 common cancer types. For each cell line, both H3K27ac ChIP-seq and ATAC-seq or deoxyribonuclease sequencing (DNase-seq) datasets were obtained from

the ENCODE project or the Gene Expression Omnibus (GEO) (data sources are listed in table S2). For cell lines with multiple biological replicates, peaks were integrated using the Irreproducible Discovery Rate (IDR) framework (*53*). An IDR threshold of 0.05 was applied following ENCODE guidelines (*54*) to retain high-confidence peaks that exhibit ≥95% reproducibility between replicates (an irreproducible fraction <5%), ensuring that only robust and consistent regulatory signals were included. For cell lines not included in ENCODE, processed peak files were downloaded from ChIP-Atlas, which applies a standardized analysis pipeline: read alignment using Bowtie2 (version 2.4.5), PCR duplicate removal with SAMtools (version 1.17), and peak calling by MACS2 (version 2.2.9.1) with a significance threshold of $q < 1 \times 10^{-5}$.

Last, the intersection of H3K27ac and ATAC/DNase peaks was defined as the putative enhancer regions for each cell line, ensuring that both histone modification and chromatin accessibility signals were present. To systematically assess the tissue specific enhancer activity, we annotated a total of 18,880 CRC enhancer regions. Using bedtools intersect (version 2.30.0), each CRC enhancer was compared with enhancer regions from the other 19 cancer types, and the number of overlaps for each enhancer was calculated. All analyses were performed on the hg19 (GRCh37) reference genome.

STARR-seq data analysis

STARR-seq reads were aligned to a custom oligonucleotide library using Bowtie2 (version 2.5.2). Read counts for each oligo were determined separately for input DNA and output RNA libraries, generating corresponding count matrices. Normalization was performed using the DESeq2 (version 1.40.0) package in R. For each oligo, an EAS was calculated as follows: EAS = normalized (RNA + 1)/normalized (DNA). For each SNP or methylation site, the fold change in enhancer activity (EAS_{alt}/EAS_{ref} or $EAS_{methylated}/EAS_{unmethylated}$) was calculated. To define this geometrically, we first sort the $\log_2$FC values in ascending order. For the right-side threshold, we reset all values less than 0 to zero. We then define the inflection point as the point where a straight line with slope is tangent to the curve [slope = $\max(\log_2\text{FC}) - \min(\log_2\text{FC})$]. Similarly, for the left-side threshold, we reset all values greater than 0 to zero and use the same slope calculation to determine the inflection point. SNPs or methylation sites exhibiting $\log_2$FC values greater than the right-side threshold or less than the left-side threshold are subsequently selected for differential analysis. Statistical significance of enhancer activity differences was assessed using a two-sided *t* test, with $P < 0.05$ considered significant.

RNA-seq data analysis

RNA-seq reads were aligned to the human reference genome (hg19) using HISAT2 (version 2.2.1). Gene-level read counts were quantified using featureCounts (version 2.0.1). Differential gene expression analysis was performed using the DESeq2 package (version 1.40.0). Genes with $\log_2\text{FC} > 0.75$ and adjusted *P* value (*P*adj) < 0.05 were considered DEGs. DEG visualization was carried out using the ggplot2 package (version 3.5.1), and GO enrichment analysis was conducted using clusterProfiler (version 4.8.3). RNA-seq datasets for the SW480 and SW620 cell lines were obtained from GEO (www.ncbi.nlm.nih.gov/geo/query/acc.cgi?acc=GSE131948).

Motif enrichment analysis of HOMER

To identify TFs potentially associated with enhancer activity changes, we used the "findMotifsGenome.pl" command in the HOMER (version 4.11) software. Differential enhancers identified from the STARR-seq assay were used as the input set and randomly selected genomic regions served as background. All analyses were performed using the hg19 reference genome.

Motif scanning with FIMO

We constructed FASTA-format sequence files containing the genomic regions surrounding each target SNP. Nonredundant TF motif files for eukaryotes were downloaded from the JASPAR CORE database (JASPAR, a database of TF-binding profiles). Motif scanning was performed using the online tool FIMO (Find Individual Motif Occurrences; https://meme-suite.org/meme/tools/fimo), which identifies statistically significant motif matches within the input sequences.

eQTL data extraction

To comprehensively assess the regulatory potential of identified SNPs, we integrated eQTL evidence from both normal and tumor contexts. Normal tissue context: Summary statistics for eQTL in normal colon tissue were obtained via the FIVEx platform (*55*) (https://fivex.sph.umich.edu/), which aggregates and serves processed data from the European Bioinformatics Institute (EBI) eQTL Catalogue (www.ebi.ac.uk/eqtl/). For each target variant, we queried the public FIVEx API endpoint (https://fivex.sph.umich.edu/) and retrieved JSON-formatted results. Results were filtered to retain only records from the sigmoid colon (colon_sigmoid) and transverse colon (colon_transverse).

Primary tumor context: TCGA-COAD and rectal adenocarcinoma (READ) eQTL datasets were obtained from PancanQTL (*56*) (https://gong_lab.hzau.edu.cn/PancanQTL). SNP was considered to have supported eQTL evidence if it was significantly associated with gene expression in either the normal colon tissue dataset or the primary tumor datasets ($P < 0.05$, FDR < 0.05). This inclusive approach allowed us to capture SNPs with broad regulatory potential across physiological states.

Construction and evaluation of 14 cancer methylation probe classification models

We obtained the TCGA 450k methylation data from UCSC Xena (https://xenabrowser.net/datapages/). After sorting and statistical analysis, we identified 14 types of cancers with at least 10 normal and tumor samples each. We extracted the methylation level data of cg08640619 and cg25982657, along with the sample labels, from the methylation data of these cancer types. Using the Python scikit library (version 3.12.10), we constructed training and testing datasets with the train_test_split function, setting the test_size parameter to 0.4 to ensure balanced representation of normal and tumor samples. We implemented two classification algorithms: LogR and SVM. To evaluate and compare their performance during the model selection phase, we used 10-fold cross-validation on the training set and assessed classification accuracy using the cross_val_score function from scikit-learn. Then, the AUROC, computed via the scikit-learn metrics module, was used as the primary metric to assess the generalization performance of the selected classifiers on unseen data. Last, we summarized and plotted the AUROC values of the LogR and SVM classifiers constructed with cg08640619 and cg25982657 for the 14 cancer types in bar charts.

Experimental statistical analysis

Statistical analysis was performed using GraphPad Prism (version 8, GraphPad Software Inc.). Experiments were repeated at least three times, and results are presented as means ± SD. *t* tests or analysis of variance (ANOVA) were used for statistical comparisons, with *P* values of <0.05 considered statistically significant.

Supplementary Materials

The PDF file includes:
Figs. S1 to S5
Legends for tables S1 to S18

Other Supplementary Material for this manuscript includes the following:
Tables S1 to S18

REFERENCES

1. B. Vogelstein, N. Papadopoulos, V. E. Velculescu, S. Zhou, L. A. Diaz Jr., K. W. Kinzler, Cancer genome landscapes. *Science* **339**, 1546–1558 (2013).
2. L. A. Hindorff, P. Sethupathy, H. A. Junkins, E. M. Ramos, J. P. Mehta, F. S. Collins, T. A. Manolio, Potential etiologic and functional implications of genome-wide association loci for human diseases and traits. *Proc. Natl. Acad. Sci. U.S.A.* **106**, 9362–9367 (2009).
3. F. Spitz, E. E. Furlong, Transcription factors: From enhancer binding to developmental control. *Nat. Rev. Genet.* **13**, 613–626 (2012).
4. I. Sur, J. Taipale, The role of enhancers in cancer. *Nat. Rev. Cancer* **16**, 483–493 (2016).
5. M. R. Corces, J. M. Granja, S. Shams, B. H. Louie, J. A. Seoane, W. Zhou, T. C. Silva, C. Groeneveld, C. K. Wong, S. W. Cho, A. T. Satpathy, M. R. Mumbach, K. A. Hoadley, A. G. Robertson, N. C. Sheffield, I. Felau, M. A. A. Castro, B. P. Berman, L. M. Staudt, J. C. Zenklusen, P. W. Laird, C. Curtis, W. J. Greenleaf, H. Y. Chang, The chromatin accessibility landscape of primary human cancers. *Science* **362**, eaav1898 (2018).
6. C. A. Boix, B. T. James, Y. P. Park, W. Meuleman, M. Kellis, Regulatory genomic circuitry of human disease loci by integrative epigenomics. *Nature* **590**, 300–307 (2021).
7. S. Tuupanen, M. Turunen, R. Lehtonen, O. Hallikas, S. Vanharanta, T. Kivioja, M. Bjorklund, G. Wei, J. Yan, I. Niittymaki, J. P. Mecklin, H. Jarvinen, A. Ristimaki, M. Di-Bernardo, P. East, L. Carvajal-Carmona, R. S. Houlston, I. Tomlinson, K. Palin, E. Ukkonen, A. Karhu, J. Taipale, L. A. Aaltonen, The common colorectal cancer predisposition SNP rs6983267 at chromosome 8q24 confers potential to enhanced Wnt signaling. *Nat. Genet.* **41**, 885–890 (2009).
8. M. M. Pomerantz, X. Qiu, Y. Zhu, D. Y. Takeda, W. Pan, S. C. Baca, A. Gusev, K. D. Korthauer, T. M. Severson, G. Ha, S. R. Viswanathan, J. H. Seo, H. M. Nguyen, B. Zhang, B. Pasaniuc, C. Giambartolomei, S. A. Alaiwi, C. A. Bell, E. P. O'Connor, M. S. Chabot, D. R. Stillman, R. Lis, A. Font-Tello, L. Li, P. Cejas, A. M. Bergman, J. Sanders, H. G. van der Poel, S. A. Gayther, K. Lawrenson, M. A. S. Fonseca, J. Reddy, R. I. Corona, G. Martovetsky, B. Egan, T. Choueiri, L. Ellis, I. P. Garraway, G. M. Lee, E. Corey, H. W. Long, W. Zwart, M. L. Freedman, Prostate cancer reactivates developmental epigenomic programs during metastatic progression. *Nat. Genet.* **52**, 790–799 (2020).
9. R. E. Bell, T. Golan, D. Sheinboim, H. Malcov, D. Amar, A. Salamon, T. Liron, S. Gelfman, Y. Gabet, R. Shamir, C. Levy, Enhancer methylation dynamics contribute to cancer plasticity and patient mortality. *Genome Res.* **26**, 601–611 (2016).
10. L. N. Kellman, P. H. Neela, S. Srinivasan, Z. Siprashvili, R. L. Shanderson, A. W. Hong, D. Rao, D. F. Porter, D. L. Reynolds, R. M. Meyers, M. G. Guo, X. Yang, Y. Zhao, G. G. Wozniak, L. K. H. Donohue, R. Shenoy, L. A. Ko, D. T. Nguyen, S. Mondal, O. S. Garcia, L. E. Elcavage, I. Elfaki, N. S. Abell, S. Tao, C. M. Lopez, S. B. Montgomery, P. A. Khavari, Functional analysis of cancer-associated germline risk variants. *Nat. Genet.* **57**, 718–728 (2025).
11. Y. Li, M. Tan, A. Akkari-Henić, L. Zhang, M. Kip, S. Sun, J. J. Sepers, N. Xu, Y. Ariyurek, S. L. Kloet, R. P. Davis, H. Mikkers, J. J. Gruber, M. P. Snyder, X. Li, B. Pang, Genome-wide Cas9-mediated screening of essential non-coding regulatory elements via libraries of paired single-guide RNAs. *Nat. Biomed. Eng.* **8**, 890–908 (2024).
12. J. Yan, Y. Qiu, A. M. Ribeiro Dos Santos, Y. Yin, Y. E. Li, N. Vinckier, N. Nariai, P. Benaglio, A. Raman, X. Li, S. Fan, J. Chiou, F. Chen, K. A. Frazer, K. J. Gaulton, M. Sander, J. Taipale, B. Ren, Systematic analysis of binding of transcription factors to noncoding variants. *Nature* **591**, 147–151 (2021).
13. C. A. Kalita, C. D. Brown, A. Freiman, J. Isherwood, X. Wen, R. Pique-Regi, F. Luca, High-throughput characterization of genetic effects on DNA-protein binding and gene transcription. *Genome Res.* **28**, 1701–1708 (2018).
14. A. J. Lea, C. M. Vockley, R. A. Johnston, C. A. Del Carpio, L. B. Barreiro, T. E. Reddy, J. Tung, Genome-wide quantification of the effects of DNA methylation on human gene regulation. *eLife* **7**, e37513 (2018).
15. A. Auton, L. D. Brooks, R. M. Durbin, E. P. Garrison, H. M. Kang, J. O. Korbel, J. L. Marchini, S. McCarthy, G. A. McVean, G. R. Abecasis, A global reference for human genetic variation. *Nature* **526**, 68–74 (2015).
16. G. L. Rampioni Vinciguerra, M. Capece, G. Scafetta, S. Rentsch, A. Vecchione, F. Lovat, C. M. Croce, Role of Fra-2 in cancer. *Cell Death Differ.* **31**, 136–149 (2024).
17. K. S. Bishnupuri, Q. Luo, N. Murmu, C. W. Houchen, S. Anant, B. K. Dieckgraefe, Reg IV activates the epidermal growth factor receptor/Akt/AP-1 signaling pathway in colon adenocarcinomas. *Gastroenterology* **130**, 137–149 (2006).
18. S. Li, X. D. Fang, X. Y. Wang, B. Y. Fei, Fos-like antigen 2 (FOSL2) promotes metastasis in colon cancer. *Exp. Cell Res.* **373**, 57–61 (2018).
19. C. Zeng, K. Matsuda, W. H. Jia, J. Chang, S. S. Kweon, Y. B. Xiang, A. Shin, S. H. Jee, D. H. Kim, B. Zhang, Q. Cai, X. Guo, J. Long, N. Wang, R. Courtney, Z. Z. Pan, C. Wu, A. Takahashi, M. H. Shin, K. Matsuo, F. Matsuda, Y. T. Gao, J. H. Oh, S. Kim, K. J. Jung, Y. O. Ahn, Z. Ren, H. L. Li, J. Wu, J. Shi, W. Wen, G. Yang, B. Li, B. T. Ji, H. Brenner, R. E. Schoen, S. Küry, S. B. Gruber, F. R. Schumacher, S. L. Stenzel, G. Casey, J. L. Hopper, M. A. Jenkins, H. R. Kim, J. Y. Jeong, J. W. Park, K. Tajima, S. H. Cho, M. Kubo, X. O. Shu, D. Lin, Y. X. Zeng, W. Zheng, Identification of susceptibility loci and genes for colorectal cancer risk. *Gastroenterology* **150**, 1633–1645 (2016).
20. B. Zhang, K. Yao, E. Zhou, L. Zhang, C. Cheng, Chr20q amplification defines a distinct molecular subtype of microsatellite stable colorectal cancer. *Cancer Res.* **81**, 1977–1987 (2021).
21. S. Säisä-Borreill, G. Davidson, T. Kleiber, A. Thevenot, E. Martin, S. Mondot, H. Blottière, A. Helleux, G. Mengus, M. Plateroti, I. Duluc, I. Davidson, J. N. Freund, General transcription factor TAF4 antagonizes epigenetic silencing by Polycomb to maintain intestine stem cell functions. *Cell Death Differ.* **30**, 839–853 (2023).
22. K. Zhao, T. Sun, Q. Sun, Z. Chen, T. Wang, J. Yang, L. Li, Y. Zhu, X. Liu, D. Yang, B. Lin, N. Lu, Nerve growth factor signaling promotes nuclear translocation of TRAF4 to enhance tumor stemness and metastatic dormancy via C-Jun-mediated IL-8 autocrine. *Adv. Sci.* **12**, e2414437 (2025).
23. S. Hu, M. Wang, A. Ji, J. Yang, R. Gao, X. Li, L. Sun, J. Wang, Y. Zhang, H. Liu, Mutant p53 and ELK1 co-drive FRA-1 expression to induce metastasis in breast cancer. *FEBS Lett.* **597**, 3087–3101 (2023).
24. L. Shu, A. Chen, L. Li, L. Yao, Y. He, J. Xu, W. Gu, Q. Li, K. Wang, T. Zhang, G. Liu, NRG1 regulates Fra-1 transcription and metastasis of triple-negative breast cancer cells via the c-Myc ubiquitination as manipulated by ERK1/2-mediated Fbxw7 phosphorylation. *Oncogene* **41**, 907–919 (2022).
25. J. Chou, M. Kaller, M. Rokavec, F. Liu, H. Hermeking, AP4 induces JNK1 and a miR-22-3p/FOSL1 feed-forward loop to activate AP-1 and promote colorectal cancer metastasis. *Cancer Commun.* **44**, 433–437 (2024).
26. W. M. Wang, A. Y. Lee, C. M. Chiang, One-step affinity tag purification of full-length recombinant human AP-1 complexes from bacterial inclusion bodies using a polycistronic expression system. *Protein Expr. Purif.* **59**, 144–152 (2008).
27. D. Song, Y. Lian, L. Zhang, The potential of activator protein 1 (AP-1) in cancer targeted therapy. *Front. Immunol.* **14**, 1224892 (2023).
28. A. Chatterjee, T. Roy, S. Swarnakar, Transcriptional upregulation of MMP-9 gene under hyperglycemic conditions in AGS cells: Role of AP-1 transcription factor. *Cell. Signal.* **124**, 111435 (2024).
29. X. Zhu, S. You, X. Du, K. Song, T. Lv, H. Zhao, Q. Yao, LRRC superfamily expression in stromal cells predicts the clinical prognosis and platinum resistance of ovarian cancer. *BMC Med. Genomics* **16**, 10 (2023).
30. P. Hingorani, M. E. Roth, Y. Wang, W. Zhang, J. B. Gill, D. J. Harrison, B. Teicher, S. Erickson, G. Gatto, M. A. Smith, E. A. Kolb, R. Gorlick, ABBV-085, antibody-drug conjugate targeting LRRC15, is effective in osteosarcoma: A report by the Pediatric Preclinical Testing Consortium. *Mol. Cancer Ther.* **20**, 535–540 (2021).
31. P. Renbaum, D. Abrahamove, A. Fainsod, G. G. Wilson, S. Rottem, A. Razin, Cloning, characterization, and expression in *Escherichia coli* of the gene coding for the CpG DNA methylase from *Spiroplasma* sp. strain MQ1(M.SssI). *Nucleic Acids Res.* **18**, 1145–1152 (1990).
32. Y. Yin, E. Morgunova, A. Jolma, E. Kaasinen, B. Sahu, S. Khund-Sayeed, P. K. Das, T. Kivioja, K. Dave, F. Zhong, K. R. Nitta, M. Taipale, A. Popov, P. A. Ginno, S. Domcke, J. Yan, D. Schübeler, C. Vinson, J. Taipale, Impact of cytosine methylation on DNA binding specificities of human transcription factors. *Science* **356**, eaaj2239 (2017).
33. E. Khosh Kish, M. Choudhry, Y. Gamallat, S. M. Buharideen, D. D, T. A. Bismar, The expression of proto-oncogene ETS-related gene (ERG) plays a central role in the oncogenic mechanism involved in the development and progression of prostate cancer. *Int. J. Mol. Sci.* **23**, 4772 (2022).
34. Y. Wang, Z. Huang, M. Sun, W. Huang, L. Xia, ETS transcription factors: Multifaceted players from cancer progression to tumor immunity. *Biochim. Biophys. Acta Rev. Cancer* **1878**, 188872 (2023).
35. Q. Luo, F. Shen, S. Zhao, L. Dong, J. Wei, H. Hu, Q. Huang, Q. Wang, P. Yang, W. Liang, W. Li, F. He, J. Cao, LINC00460/miR-186-3p/MYC feedback loop facilitates colorectal cancer immune escape by enhancing CD47 and PD-L1 expressions. *J. Exp. Clin. Cancer Res.* **43**, 225 (2024).
36. P. Hou, S. Meng, M. Li, T. Lin, S. Chu, Z. Li, J. Zheng, Y. Gu, J. Bai, LINC00460/DHX9/IGF2BP2 complex promotes colorectal cancer proliferation and metastasis by mediating HMGA1 mRNA stability depending on m6A modification. *J. Exp. Clin. Cancer Res.* **40**, 52 (2021).
37. Y. Lian, C. Yan, H. Xu, J. Yang, Y. Yu, J. Zhou, Y. Shi, J. Ren, G. Ji, K. Wang, A novel lncRNA, LINC00460, affects cell proliferation and apoptosis by regulating KLF2 and CUL4A expression in colorectal cancer. *Mol. Ther. Nucleic Acids* **12**, 684–697 (2018).
38. H. Zhang, Y. Lu, J. Wu, J. Feng, LINC00460 hypomethylation promotes metastasis in colorectal carcinoma. *Front. Genet.* **10**, 880 (2019).

39. C. C. Lee, W. S. Chen, C. C. Chen, L. L. Chen, Y. S. Lin, C. S. Fan, T. S. Huang, TCF12 protein functions as transcriptional repressor of E-cadherin, and its overexpression is correlated with metastasis of colorectal cancer. *J. Biol. Chem.* **287**, 2798–2809 (2012).
40. S. Teng, Y. E. Li, M. Yang, R. Qi, Y. Huang, Q. Wang, Y. Zhang, S. Chen, S. Li, K. Lin, Y. Cao, Q. Ji, Q. Gu, Y. Cheng, Z. Chang, W. Guo, P. Wang, I. Garcia-Bassets, Z. J. Lu, D. Wang, Tissue-specific transcription reprogramming promotes liver metastasis of colorectal cancer. *Cell Res.* **30**, 34–49 (2020).
41. A. Melnikov, A. Murugan, X. Zhang, T. Tesileanu, L. Wang, P. Rogov, S. Feizi, A. Gnirke, C. G. Callan Jr., J. B. Kinney, M. Kellis, E. S. Lander, T. S. Mikkelsen, Systematic dissection and optimization of inducible enhancers in human cells using a massively parallel reporter assay. *Nat. Biotechnol.* **30**, 271–277 (2012).
42. A. Melnikov, X. Zhang, P. Rogov, L. Wang, T. S. Mikkelsen, Massively parallel reporter assays in cultured mammalian cells. *J. Vis. Exp.* **90**, 10.3791/51719 (2014).
43. S. Liu, Y. Liu, Q. Zhang, J. Wu, J. Liang, S. Yu, G. H. Wei, K. P. White, X. Wang, Systematic identification of regulatory variants associated with cancer risk. *Genome Biol.* **18**, 194 (2017).
44. C. D. Arnold, D. Gerlach, C. Stelzer, Ł. M. Boryń, M. Rath, A. Stark, Genome-wide quantitative enhancer activity maps identified by STARR-seq. *Science* **339**, 1074–1077 (2013).
45. J. C. Ulirsch, S. K. Nandakumar, L. Wang, F. C. Giani, X. Zhang, P. Rogov, A. Melnikov, P. McDonel, R. Do, T. S. Mikkelsen, V. G. Sankaran, Systematic functional dissection of common genetic variation affecting red blood cell traits. *Cell* **165**, 1530–1545 (2016).
46. P. J. Law, J. Studd, J. Smith, J. Vijayakrishnan, B. T. Harris, M. Mandelia, C. Mills, M. G. Dunlop, R. S. Houlston, Systematic prioritization of functional variants and effector genes underlying colorectal cancer risk. *Nat. Genet.* **56**, 2104–2111 (2024).
47. D. C. Bruhm, N. A. Vulpescu, Z. H. Foda, J. Phallen, R. B. Scharpf, V. E. Velculescu, Genomic and fragmentomic landscapes of cell-free DNA for early cancer detection. *Nat. Rev. Cancer* **25**, 341–358 (2025).
48. G. Jung, E. Hernández-Illán, L. Moreira, F. Balaguer, A. Goel, Epigenetics of colorectal cancer: Biomarker and therapeutic potential. *Nat. Rev. Gastroenterol. Hepatol.* **17**, 111–130 (2020).
49. Q. Cao, Y. Tian, Z. Deng, F. Yang, E. Chen, Epigenetic alteration in colorectal cancer: Potential diagnostic and prognostic implications. *Int. J. Mol. Sci.* **25**, 3358 (2024).
50. J. H. Souverijn, Multitarget stool DNA testing for colorectal-cancer screening. *N. Engl. J. Med.* **371**, 187 (2014).
51. T. R. Church, M. Wandell, C. Lofton-Day, S. J. Mongin, M. Burger, S. R. Payne, E. Castaños-Vélez, B. A. Blumenstein, T. Rösch, N. Osborn, D. Snover, R. W. Day, D. F. Ransohoff, Prospective evaluation of methylated SEPT9 in plasma for detection of asymptomatic colorectal cancer. *Gut* **63**, 317–325 (2014).
52. H. Luo, W. Wei, Z. Ye, J. Zheng, R. H. Xu, Liquid biopsy of methylation biomarkers in cell-free DNA. *Trends Mol. Med.* **27**, 482–500 (2021).
53. Q. Li, J. B. Brown, H. Huang, P. J. Bickel, Measuring reproducibility of high-throughput experiments. *Ann. Appl. Stat.* **5**, 1752–1779 (2011).
54. S. G. Landt, G. K. Marinov, A. Kundaje, P. Kheradpour, F. Pauli, S. Batzoglou, B. E. Bernstein, P. Bickel, J. B. Brown, P. Cayting, Y. Chen, G. DeSalvo, C. Epstein, K. I. Fisher-Aylor, G. Euskirchen, M. Gerstein, J. Gertz, A. J. Hartemink, M. M. Hoffman, V. R. Iyer, Y. L. Jung, S. Karmakar, M. Kellis, P. V. Kharchenko, Q. Li, T. Liu, X. S. Liu, L. Ma, A. Milosavljevic, R. M. Myers, P. J. Park, M. J. Pazin, M. D. Perry, D. Raha, T. E. Reddy, J. Rozowsky, N. Shoresh, A. Sidow, M. Slattery, J. A. Stamatoyannopoulos, M. Y. Tolstorukov, K. P. White, S. Xi, P. J. Farnham, J. D. Lieb, B. J. Wold, M. Snyder, ChIP-seq guidelines and practices of the ENCODE and modENCODE consortia. *Genome Res.* **22**, 1813–1831 (2012).
55. A. Kwong, A. P. Boughton, M. Wang, P. VandeHaar, M. Boehnke, G. Abecasis, H. M. Kang, FIVEx: An interactive eQTL browser across public datasets. *Bioinformatics* **38**, 559–561 (2022).
56. J. Gong, S. Mei, C. Liu, Y. Xiang, Y. Ye, Z. Zhang, J. Feng, R. Liu, L. Diao, A.-Y. Guo, X. Miao, L. Han, PancanQTL: Systematic identification of cis-eQTLs and trans-eQTLs in 33 cancer types. *Nucleic Acids Res.* **46**, D971–D976 (2018).

Acknowledgments: We are grateful to M. Lai, Y. Zhao, Y. Li, Y. Ren, and X. Yu, the undergraduates from School of Medicine, Northwest University, for invaluable technical contributions to the work. **Funding:** This work was supported by Shenzhen Medical Research Fund (grant no. B2302027), the National Natural Science Foundation of China (grant nos. 32270634 and 82003115), the Shaanxi Science and Technology Innovation Team Project (2024RS-CXTD-85), the Shaanxi Academy of Fundamental Sciences (Chemistry and Biology) (grant no. 22JHZ009), Natural Science Foundation of Guangdong Province (grant no. 2024A1515012685), the Research Grants Council of Hong Kong (grant no. 11101022), Innovation and Technology Commission of Hong Kong (ITS/087/22), and City University of Hong Kong (9609317 and 7006043). **Author contributions:** Conceptualization: E.C. and J.Y. Project administration: E.C. and J.Y. Investigation: R.H., Y.C., Q.W., H.D., E.C., J.W., and M.C. Methodology: E.C., J.Y., and Y.Z. Formal analysis: Q.X., Y.Z., and W.S. Visualization: Q.X., Q.Y., E.C., J.Y., Y.Z., and W.S. Validation: L.F., E.C., and Y.-Q.N. Data curation: Q.W., Q.Y., H.D., E.C., and M.C. Writing—original draft: Q.W., Q.Y., H.D., E.C., and J.Y. Writing—review and editing: E.C., J.Y. Supervision: L.F., E.C., Y.-Q.N., and J.Y. Resources: E.C. and J.Y. Funding acquisition: E.C., L.F., and J.Y. **Competing interests:** The authors declare that they have no competing interests. **Data, code, and materials availability:** The raw sequence data reported in this paper have been deposited in the Genome Sequence Archive (Genomics, Proteomics & Bioinformatics, 2021) in National Genomics Data Center (Nucleic Acids Res., 2024), China National Center for Bioinformation/Beijing Institute of Genomics, Chinese Academy of Sciences (GSA-Human: HRA012284) that are publicly accessible at https://ngdc.cncb.ac.cn/gsa-human. The epigenomic datasets for representative cell lines from 20 common cancer types from the ENCODE and the Gene Expression Omnibus (GEO) and the summary of data source are available in table S2. The eQTL summary statistics for eQTL in normal colon tissue were obtained via the FIVEx platform (https://fivex.sph.umich.edu/), which aggregates and serves processed data from the European Bioinformatics Institute (EBI) eQTL Catalogue (www.ebi.ac.uk/eqtl/). TCGA-COAD and READ eQTL datasets were obtained from PancanQTL (https://gong_lab.hzau.edu.cn/PancanQTL/download). RNA-seq datasets for the SW480 and SW620 cell lines were downloaded from the GEO (www.ncbi.nlm.nih.gov/geo/query/acc.cgi?acc=GSE131948). Genome-wide H3K27ac histone modification profiles for SW480 and SW620 cell lines were downloaded from the GEO seriesGSE136888 (www.ncbi.nlm.nih.gov/geo/query/acc.cgi?acc=GSE136888). All primer sequences used in this study are available in tables S12 to S17. All data and code needed to evaluate and reproduce the results in the paper are present in the paper and/or the Supplementary Materials.

Submitted 6 August 2025
Accepted 13 January 2026
Published 20 February 2026
10.1126/sciadv.aeb2473
