# Supplementary material for: Systematic analysis of functional genetic and epigenetic variants in colorectal cancer
Source: Sci Adv. 2026 Feb 20;12(8):eaeb2473. doi: 10.1126/sciadv.aeb2473 (PMC12922747; doi:10.1126/sciadv.aeb2473)
Supplement: Supplementary file 2 — Figs. S1 to S5 Legends for tables S1 to S18 [file sciadv.aeb2473_sm.pdf]

Science Advances
AAAS

Supplementary Materials for

Systematic analysis of functional genetic and epigenetic variants in colorectal cancer

Erfei Chen *et al.*

Corresponding author: Jian Yan, jian.yan@cityu.edu.hk

Sci. Adv. **12**, eaeb2473 (2026)
DOI: 10.1126/sciadv.aeb2473

The PDF file includes:

Figs. S1 to S5
Legends for tables S1 to S18

Other Supplementary Material for this manuscript includes the following:

Tables S1 to S18

Chen et al. Supplementary fig. S1

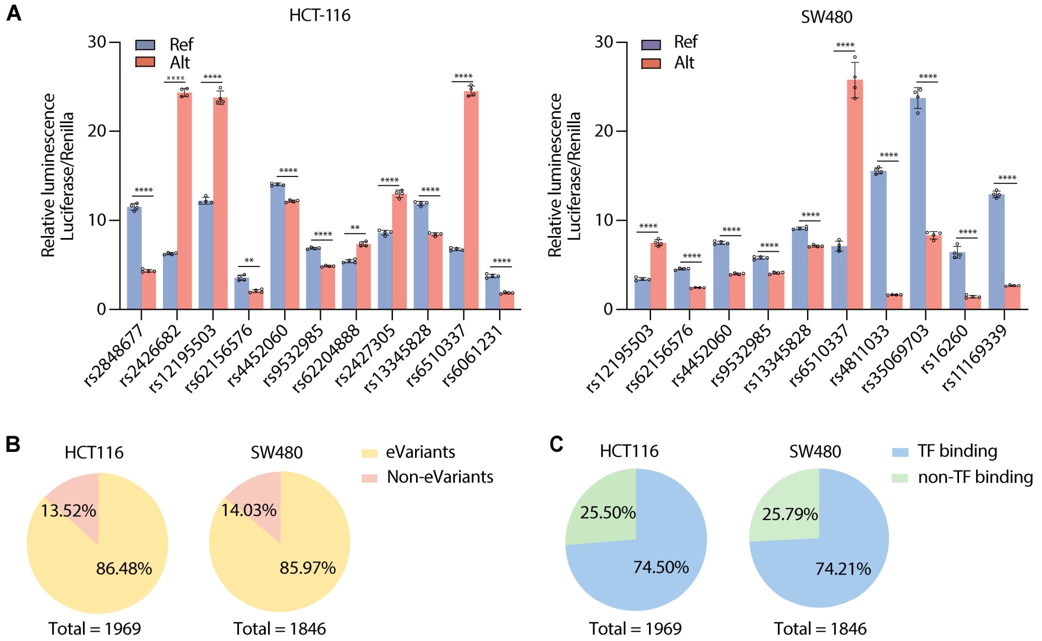

D

Top 10 motifs enriched in HCT116

Rank	Motif	Consensus	P-value	%sites	q-value
1	Fosl2		10^{-29}	6.20	$<10^{-4}$
2	Fra2		10^{-26}	7.26	$<10^{-4}$
3	Fra1		10^{-26}	7.82	$<10^{-4}$
4	JUN-AP1		10^{-26}	4.93	$<10^{-4}$
5	JUNB		10^{-26}	7.87	$<10^{-4}$
6	Fos		10^{-25}	8.13	$<10^{-4}$
7	Atf3		10^{-24}	8.68	$<10^{-4}$
8	BATF		10^{-24}	8.43	$<10^{-4}$
9	KLF1		10^{-18}	27.43	$<10^{-4}$
10	Sp1		10^{-18}	15.59	$<10^{-4}$

Top 10 motifs enriched in SW480

Rank	Motif	Consensus	P-value	%sites	q-value
1	Fra2		10^{-29}	8.07	$<10^{-4}$
2	Fosl2		10^{-27}	6.28	$<10^{-4}$
3	JUN-AP1		10^{-26}	5.20	$<10^{-4}$
4	Fra1		10^{-26}	8.40	$<10^{-4}$
5	JUNB		10^{-24}	8.29	$<10^{-4}$
6	Atf3		10^{-24}	9.37	$<10^{-4}$
7	Fos		10^{-22}	8.34	$<10^{-4}$
8	BATF		10^{-22}	9.05	$<10^{-4}$
9	Bach2		10^{-14}	3.30	$<10^{-4}$
10	NF-E2		10^{-12}	1.63	$<10^{-4}$

Figure S1. SNP-STARR-seq Identifies SNPs with allele-specific enhancer activities in different types of CRC cells.

A) Validation of allele-specific enhancer activity by dual-luciferase reporter assay. Bar charts compare the normalized luciferase activity between the reference (Ref) and alternative (Alt) alleles for selected SNPs. Data are from four biological replicates (n=4) and shown for (Left) HCT116 and (Right) SW480 cells. B) The pie charts display the percentage of paSNPs with eQTL data in HCT116 and SW480. C)The pie charts display the percentage of paSNPs with TF binding in HCT116 and SW480. D)The top 10 transcription factor motif enriched in paSNPs of HCT116 and SW480, respectively. $^{**}P<0.01$, and $^{****}P<0.0001$.

Chen et al. Supplementary fig. S2

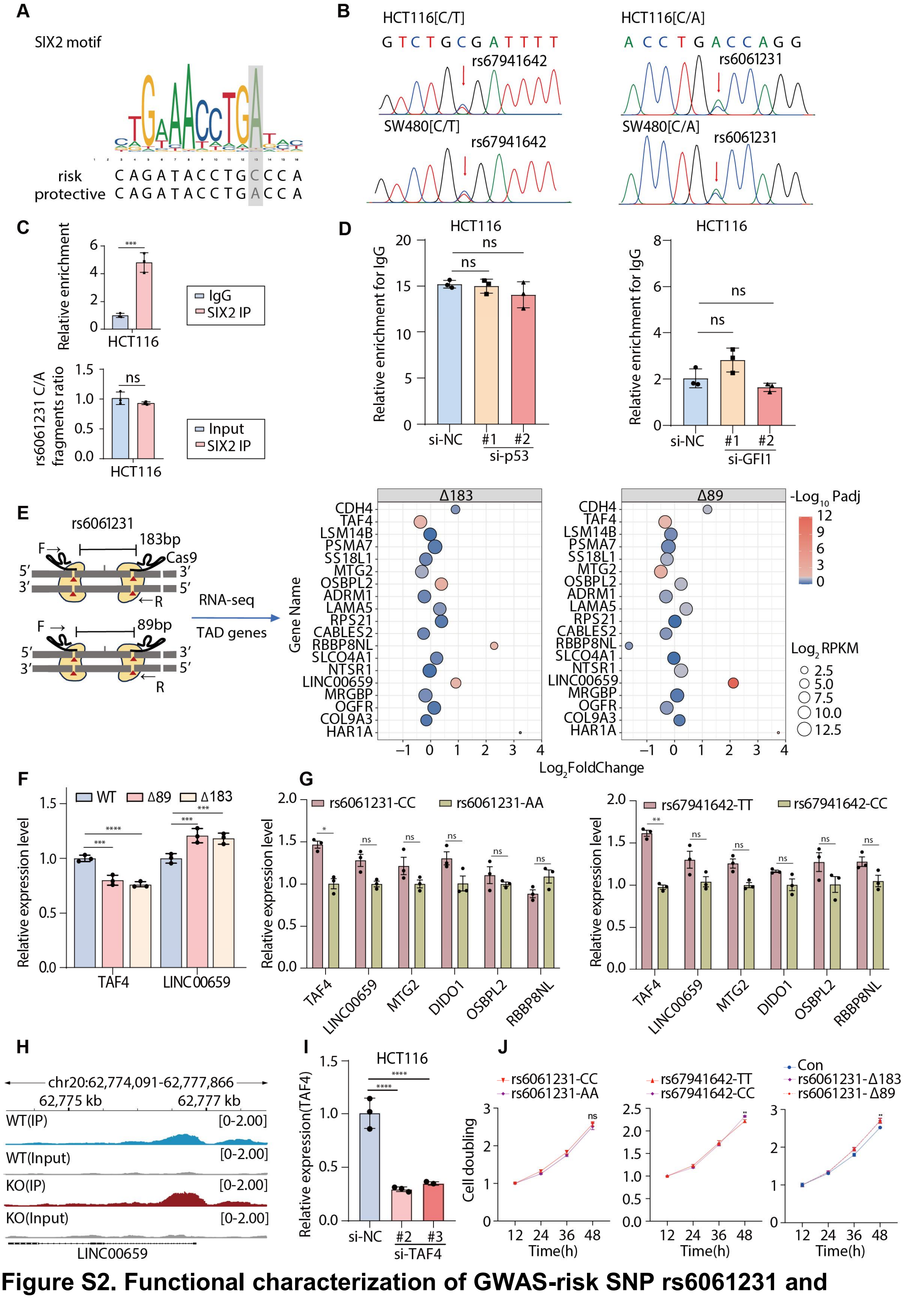

Figure S2. Functional characterization of GWAS-risk SNP rs6061231 and rs67941642.

A) Motif analysis of SIX2 at rs6061231. B) Genotyping assays in HCT116 and SW480 cells identified rs6061231 as C/A heterozygous and rs67941642 as C/T heterozygous. C) ChIP-qPCR for SIX2 binding at rs6061231 (upper panel). Allele-specific binding of SIX2 to rs6061231 was assessed by ChIP-amplicon sequencing analysis (lower

panel). D) siRNA-mediated depletion of p53 or GFI1 was performed to monitor changes in GFI1 enrichment at rs67941642 (left panel) and p53 occupancy at rs6061231 (right panel) using ChIP-qPCR. E) CRISPR-Cas9-mediated knockout of rs6061231 in HCT116 cells was generated, followed by RNA-sequencing to identify differentially expressed genes (DEGs). The circle size represents log2(RPKM) of control cells, the horizontal axis represents the log2Foldchange of fragment knockout vs control, and the color represents -log10 Padj value. Δ89: 89bp enhancer deletion cells, Δ183: 183bp enhancer deletion cells. F) Relative mRNA expression levels of TAF4 and LINC00659 following CRISPR-mediated rs6061231 deletion. △89: 89bp enhancer deletion cells, Δ183: 183bp enhancer deletion cells. G) qPCR analysis of gene expression in isogenic point-mutated HCT116 cells (Paired t test). *P<0.05, **P<0.01. H) IGV tracks of H3K27ac ChIP-seq show increased signal in the highlighted region, with the normalized enrichment ratio 3.70 in WT cells and 4.74 in KO cells. WT, wildtype HCT116 cells; KO, HCT116 cells with enhancer knockout (△89 clone). I) siRNA targeting TAF4 achieved efficient knockdown (>70%) in wild-type HCT116 cells. J) Cell proliferation analysis upon enhancer deletion and SNP point mutation. Con: cells transfected with non-targeting gRNA (NT-gRNA). Δ89: 89bp enhancer deletion cells, Δ183: 183bp enhancer deletion cells. Data points represent the mean ± SD of replicate measurements. *P<0.05, **P<0.01, ***P < 0.001, ****P < 0.0001.

Chen et al. Supplementary fig. S3

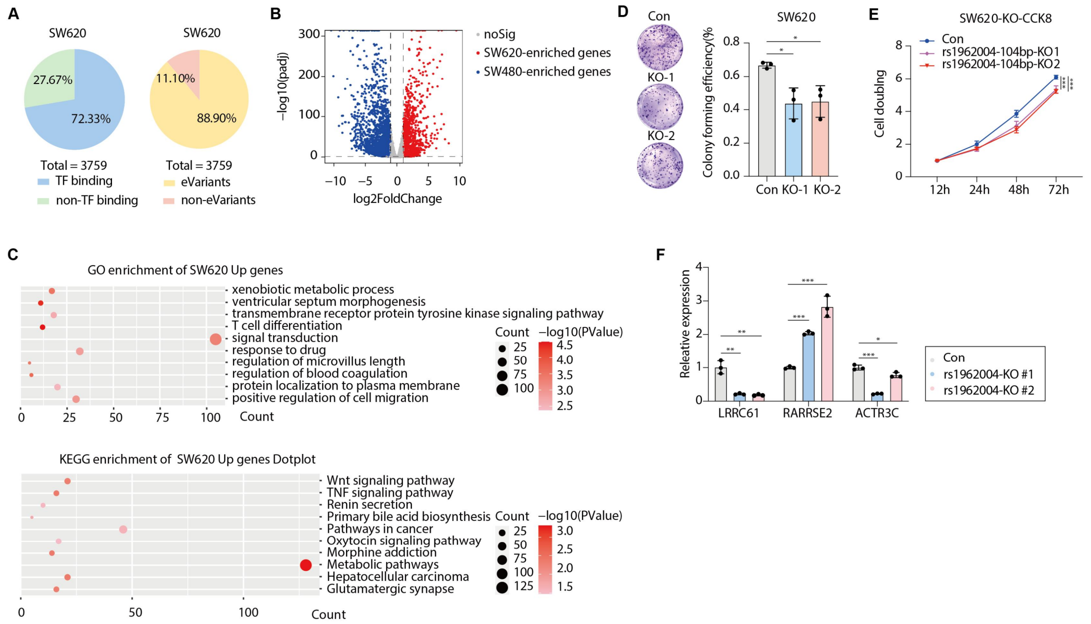

Figure S3. Identification of a functional metastasis-related SNP rs1962004. A) 3759 SNPs with activity differences were identified in SW620 cells through STARR-seq screening, and their proportions were analyzed based on transcription factor binding site information and eQTL database inclusion. B) Differentially expressed genes (DEGs) between SW620 and SW480 cell lines were identified by RNA-seq analysis ($|\log_2 FC| \geqslant 0.75$, FDR < 0.05). C) GO and KEGG enrichment of genes that were significantly up-regulated in SW620 compared to SW480 in RNA-seq. D) Knockout the 104bp region of rs1962004 in SW620 cells and demonstrate its proliferative ability through colony formation. Con: cells transfected with non-targeting gRNA (NT-gRNA) vector. E) Knockout of the 104bp fragment containing rs1962004 inhibits the proliferation of SW620. Con: cells transfected with non-targeting gRNA (NT-gRNA) vector. F) Knockout of the 104bp fragment containing rs1962004 significantly inhibited the expression of *LRRC61* (*LRRC61*, *RARRSE2*, *ACTR3C* are in the same TAD region as rs1962004). Control: cells transfected with non-targeting gRNA (NT-gRNA) vector. *P < 0.05, **P < 0.01, and ***P < 0.001.

Chen et al. Supplementary fig. S4

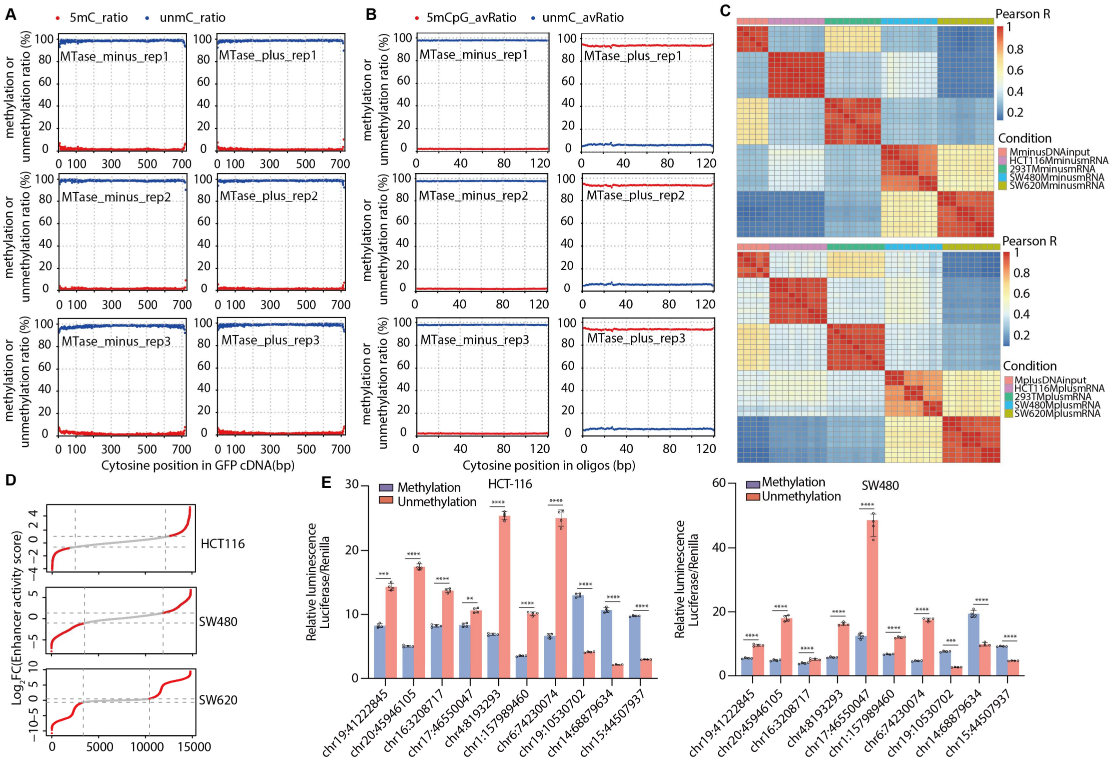

Figure S4. Methyl-STARR-seq identifies methylation-dependent enhancer elements.

A) Assessment of bisulfite conversion efficiency for BS-seq. Unmethylated GFP fragments were spiked into methylated and unmethylated control libraries, respectively. Three biological replicates showed >99.5% conversion of unmethylated cytosines to uracils following bisulfite treatment. B) BS-seq analysis of the average methylation level across the inserted fragment. The x-axis represents position along the 120-nt oligo; the y-axis shows the average methylation ratio (%) of CpG sites at each position. Following M.SssI treatment, the vast majority of CpG sites were methylated (right panel). C) Heatmap displaying Pearson correlation coefficients of normalized read counts between all biological and technical replicates for both DNA input and RNA output libraries. High correlation among DNA input replicates reflects technical reproducibility. Clustering of RNA replicates by cell type indicates robust, cell-type-specific capture of enhancer activity. The upper panel represents the mock-treated (unmethylated) control, while the lower panel represents the M.SssI-treated (methylated) condition. D) Ranking of the log2-fold changes of alt/ref enhancer activity scores. Dashed lines show the cutoff of methylated sites with significant activity changes (HCT116: left: -0.71, right: 1.10; SW480: left: -1.10, right: 1.33; SW620: left: -0.88, right: 1.95). E) Validation of methylation-dependent enhancer activity by dual-luciferase reporter assay. Bar charts compare the normalized luciferase activity between the unmethylated (Unmeth.) and methylated (Meth.) states for selected CpG-containing fragments. Data are from four biological replicates (n=4) and shown for (Left) HCT116 and (Right) SW480 cells. ***P* < 0.01, and *****P* < 0.0001.

Chen et al. Supplementary fig. S5

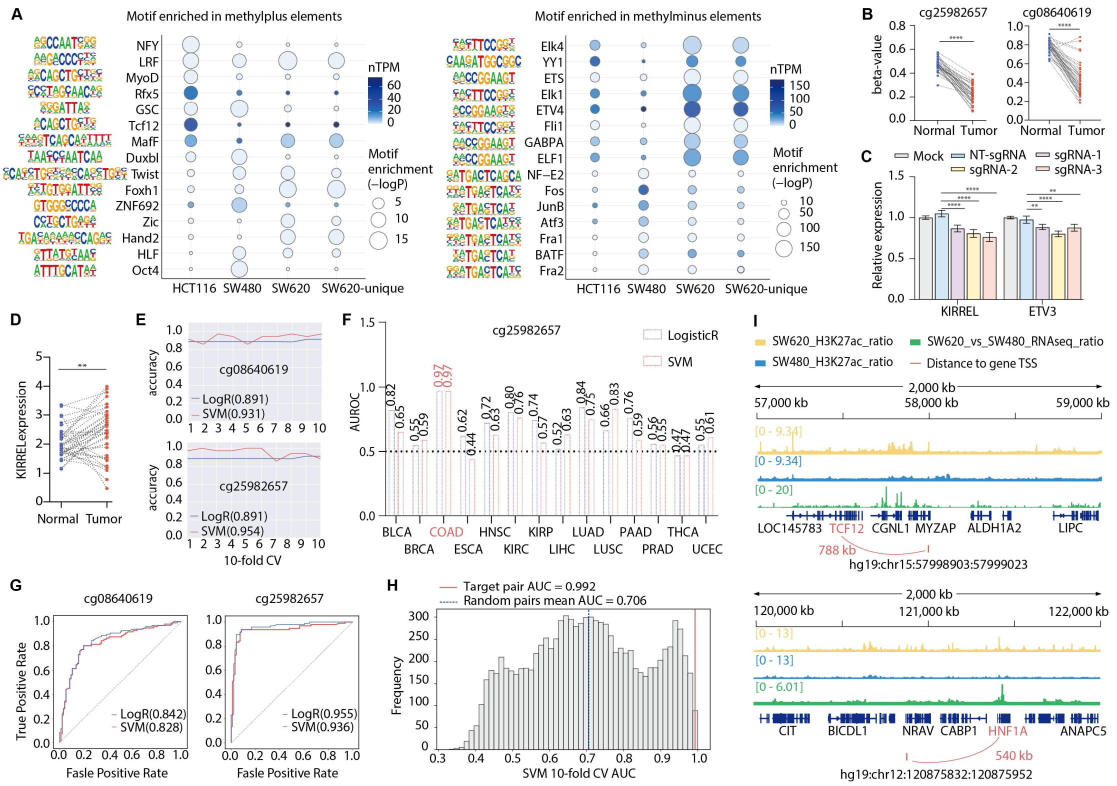

Figure S5. Methyl-STARR-seq identifies methylation-dependent enhancer elements

A) Transcription factor (TF) motif enrichment analysis in HCT116, SW480 and SW620 cell lines. Motifs were identified using HOMER by comparing regions identified as "methylplus" against those identified as "methylminus", with each set serving as the background for the other. B) Methylation analysis of CpG sites cg08640619 and cg25982657 in TCGA-COAD paired tumor/normal samples. Significance was assessed using a paired t-test. C) qPCR analysis of KIRREL and ETV3 gene expression in HCT116 cells following CRISPR-dCas9-DNMT3A-mediated epigenetic editing. D) Expression analysis of KIRREL in TCGA-COAD paired tumor/normal samples. Significance was assessed using a paired t-test (***P*=0.0084). E) Comparison of classification accuracy between Logistic Regression (LogR) and Support Vector Machine (SVM) models using 10-fold stratified cross-validation for the two CpG sites cg08640619 and cg25982657 in TCGA COAD. Each of the ten points corresponds to the accuracy achieved on an individual held-out fold, and the mean accuracy is indicated in the legend. This plot illustrates the fold-to-fold stability and relative performance of the two models. F) Bar plot displaying AUROC values for cancer classification using methylation at CpG site cg25982657, comparing Logistic Regression (LR) and Support Vector Machine (SVM) classifiers across 14 cancer types. G) Independent validation of DNA methylation-based classifiers on the GSE77718 cohort. (left) cg08640619 and (right) cg25982657. The Area Under the Curve (AUC) values for each model are indicated in the legend. H) Random background analysis to evaluate model specificity. We randomly sampled 10,000 CpG pairs and trained identical SVM models (10-fold CV) for each pair. The AUC of our

two-CpG model (0.992) exceeded 99.6% of random CpG pairs. I) IGV tracks display the metastasis-associated locus and its target gene, which is transcriptionally upregulated during metastatic progression. $^{**}P<0.01$, and $^{****}P < 0.0001$.

Supplementary Table Files:

Table S1. Genetic and Epigenetic Variants within Colorectal Cancer Enhancer Regions and Differential Enhancer Activity Identified by STARR-seq

Table S2. ATAC-seq and H3K27ac ChIP-seq data source of 20 tumor types

Table S3. SNP-STARR-seq results in 4 cell lines

Table S4. Methyl-STARR-seq results in 4 cell lines

Table S5. Detail information of 922 common SNPs identified in both HCT116 and SW480

Table S6. Detail information of 3136 metastasis-related SNPs

Table S7. Differential expressed genes in SW620/SW480 RNA-seq

Table S8. TF motif analysis of methylation sensitive loci in 3 cell lines

Table S9. 487 common methylation sensitive loci identified in both HCT116 and SW480

Table S10. The differential methylated analysis in 14 tumor types of 6 candidates

Table S11. Deatil information of 3008 CRC metastatic methyl sites

Table S12. Primers used in SNP-STARR-seq and Methyl-STARR-seq

Table S13. The cloning primers as well as the sequences of detected variant sites

Table S14. Original data for luciferase reporter assays

Table S15. PCR and sequencing primers for genotyping

Table S16. sgRNA and ssODN sequences

Table S17. siRNA and shRNA sequences

Table S18. qPCR primer sequences
